# Auxin-salicylic acid seesaw regulates the age-dependent balance between plant growth and herbivore defense

**DOI:** 10.1126/sciadv.adu5141

**Published:** 2025-05-09

**Authors:** Wen-Hao Han, Feng-Bin Zhang, Shun-Xia Ji, Kai-Lu Liang, Jun-Xia Wang, Xiao-Ping Fan, Shu-Sheng Liu, Xiao-Wei Wang

**Affiliations:** ^1^ State Key Laboratory of Rice Biology and Breeding, Ministry of Agriculture and Rural Affairs Key Lab of Molecular Biology of Crop Pathogens and Insect Pests, Zhejiang Key Laboratory of Biology and Ecological Regulation of Crop Pathogens and Insects, Institute of Insect Sciences, Zhejiang University, Hangzhou 310058, China.; ^2^ LC-Bio Technology Co. Ltd., Hangzhou 310018, China.

## Abstract

According to the plant vigor hypothesis, younger, more vigorous plants tend to be more susceptible to herbivores compared to older, mature plants, yet the molecular mechanisms underlying this dynamic remain elusive. Here, we uncover a hormonal cross-talk framework that orchestrates the age-related balance between plant growth and herbivore defense. We demonstrate that the accumulation of salicylic acid (SA), synthesized by *Nicotiana benthamiana* phenylalanine ammonia-lyase 6 (NbPAL6), dictates insect resistance in adult plants. *NbPAL6* expression is driven by the key transcription factor, NbMYB42, which is regulated by two interacting auxin response factors, NbARF18La/b. In juvenile plants, higher auxin levels activate *Nb*miR160c, a microRNA that simultaneously silences *NbARF18La/b*, subsequently reducing *NbMYB42* expression, lowering SA accumulation, and thus weakening herbivore defense. Excessive SA in juvenile plants enhances defense but antagonizes auxin signaling, impairing early growth. Our findings suggest a seesaw-like model that balances growth and defense depending on the plant’s developmental stage.

## Teaser

Phytohormone cross-talk balances growth and insect defense across growth stages.

## INTRODUCTION

Plants, being sessile organisms, must constantly navigate hostile environments to survive and reproduce, facing a wide range of abiotic and biotic stresses. Over time, they have evolved sophisticated systems to detect, respond to, and defend against environmental changes. However, these defenses come at a notable cost, often resulting in trade-offs with growth and development (*1*, *2*). Among the most damaging threats to plants are herbivorous insects, which can attack at any stage of plant development. While tissue-chewing insects cause direct damage by consuming plant tissues, phloem-feeding insects pierce the epidermis to delicately extract nutrient-rich phloem sap using needle-like mouthparts. In response to such threats, plants have evolved both inducible and constitutive defense mechanisms (*3*, *4*). Induced defenses are triggered in response to specific stimuli, while constitutive defenses involve continuous accumulation of insecticidal components throughout the plant’s growth and development (*5*, *6*). Given that insect attacks are often sporadic, plants must finely balance their investment in growth with the need for defense. However, how this balance is maintained at the molecular level remains poorly understood.

In the context of plant-herbivore interactions, the plant vigor hypothesis (PVH) posits that herbivores preferentially target young, vigorous plants over older, mature ones (*7*, *8*). At the same time, plants tend to develop increased resistance to biological stresses, such as pathogens and herbivores, as they age—a phenomenon known as age-related resistance (ARR) (*9*–*13*). These observations suggest that plants may prioritize rapid growth early in life, tolerating some degree of herbivore damage, before shifting their focus to defense mechanisms later in development (*14*). However, the precise mechanisms underlying PVH and ARR in the context of herbivores remain largely unexplored. Given the dual necessity for plants to grow and defend themselves, a better understanding of how this balance is optimized holds significance to both ecological theory and agricultural production.

Phytohormones play a pivotal role in regulating various aspects of plant growth, development, and defense, often through intricate cross-talk mechanisms (*15*, *16*). Auxin, the first phytohormone found, along with other phytohormones, orchestrates plant growth and development while also modulating stress responses (*16*). Among stress-related phytohormones, jasmonic acid (JA), salicylic acid (SA), abscisic acid, and ethylene (ET) are key players, particularly in response to herbivory, with JA being central to resistance against tissue-chewing insects and SA being crucial for defense against phloem-feeding insects (*14*, *15*). The interaction between these phytohormones has complex effects on plants against insects (*15*). For instance, the JA-SA antagonistic relationship is a well-studied area, with each hormone modulating plant responses to different herbivores. Moreover, other phytohormones, including auxin, ET, abscisic acid, gibberellins, brassinosteroids, and cytokinins are also involved in fine-tuning plant immunity by influencing JA and SA signaling pathways, especially in response to pathogens and abiotic stresses (*15*). The intricate mechanisms of phytohormonal interplay involve multiple layers of regulation, including transcriptional control by transcription factors (TFs), posttranscriptional regulation by small RNAs, and control of protein activity (*17*–*20*). Despite these advances, it remains unclear how plants integrate hormonal signals to balance growth and defense at different developmental stages.

The whitefly, *Bemisia tabaci* (Gennadius), is a species complex, several species of which are of agricultural importance worldwide (*21*–*24*). A few species of this whitefly complex are highly polyphagous, exhibiting remarkable adaptability to different hosts (*25*, *26*). Being phloem-feeders, whiteflies use their piercing-sucking mouthparts delicately to extract nutrients, making juvenile, vigorous plants particularly vulnerable to attacks and yield losses (*25*, *27*, *28*). Understanding the underlying mechanisms that govern plant growth-defense (G-D) balance could be key to developing sustainable whitefly control strategies.

*Nicotiana benthamiana* has become an important model organism in plant molecular biology and biotechnology, serving as a bio-factory for vaccines and pharmacological compounds (*29*, *30*). Our previous studies have shown that *N. benthamiana* exhibits strong attraction to and lethal effects on several Hemiptera and Thysanoptera insects, including whiteflies, winged aphids, and thrips, highlighting its potential as a dead-end trap plant for field pest control (*31*–*33*). However, we observed that the insecticidal efficacy of *N. benthamiana* varied with the plant’s development stage. Upon further investigation, we found that beyond environmental influences, this age-related defense capacity was closely tied to the developmental stage, suggesting a complex regulatory mechanism involving ARR. These findings underscore the utility of *N. benthamiana* as a system to study ARR and provide a foundation for its application in pest management.

In this study, we demonstrate that auxin-SA cross-talk mediates the balance between plant growth and herbivore defense at different developmental stages. We reveal that the establishment of ARR in *N. benthamiana* and several other Solanaceae species is closely tied to SA levels. Using molecular biology and biochemical approaches, we elucidate that the NbARF18La/b-NbMYB42-NbPAL6 signaling module plays a critical role in the age-related regulation of SA accumulation. Furthermore, we find that the age-dependent auxin–microRNA (miRNA) 160c module also contributes to this process. Our findings provide previously unknown insights into PVH and lay the theoretical groundwork for developing innovative pest control strategies against whiteflies.

## RESULTS

### Age-regulated resistance against phloem-feeding insects in Solanaceae plants

**Fig. 1.**
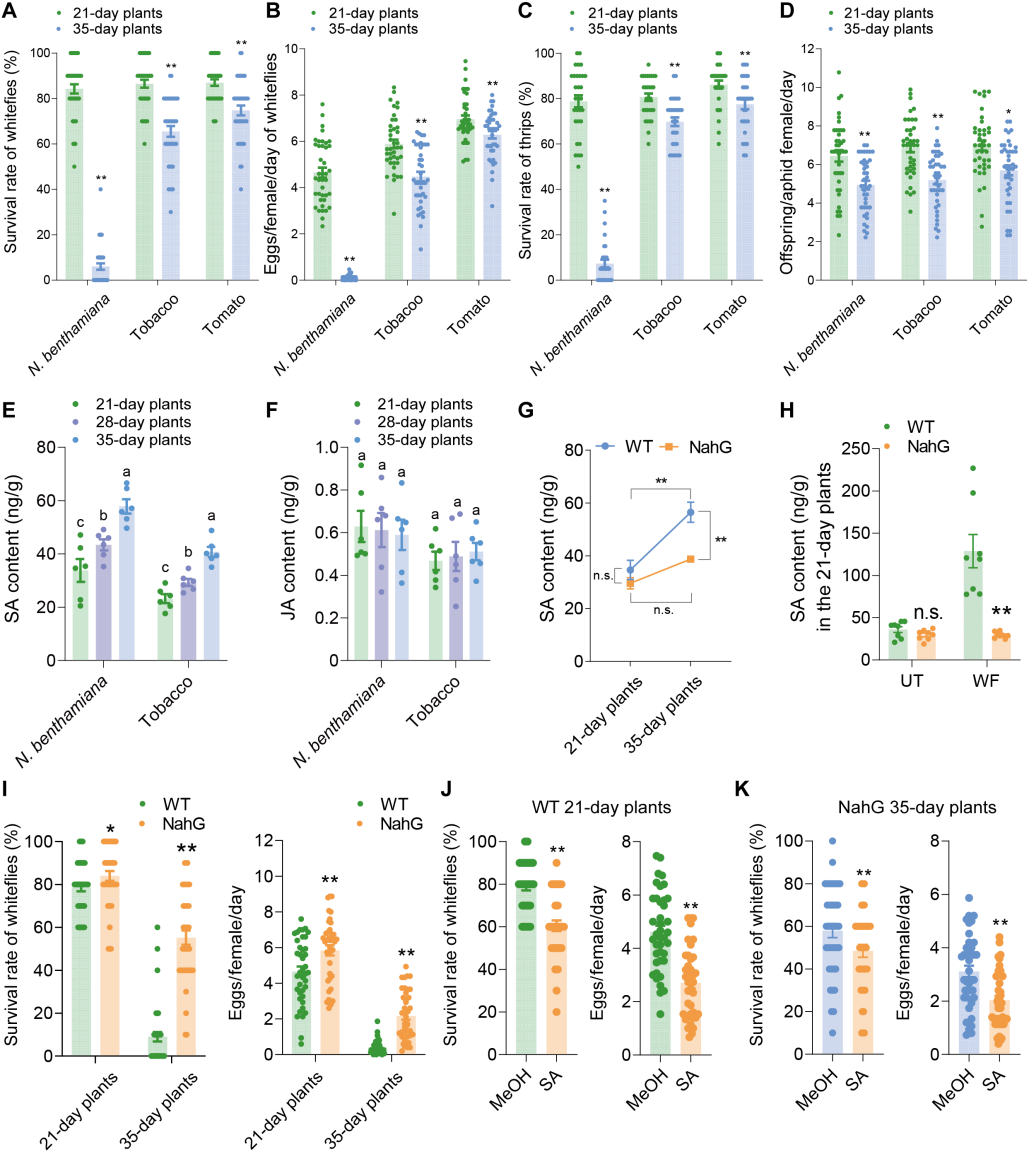
Plant ARR against insects is regulated by SA. (**A** and **B**) Survival rate and fecundity of whiteflies on 21- and 35-day-old *N. benthamiana*, tobacco, and tomato plants. (**C**) Survival rate of thrips on 21- and 35-day-old *N. benthamiana*, tobacco, and tomato plants. (**D**) Fecundity of aphids on *N. benthamiana*, tobacco, and tomato plants at 21- and 35-day ages. (**E** and **F**) SA and JA levels in *N. benthamiana* and tobacco at 21, 28, and 35 days of age. (**G**) Temporal dynamics of SA content in wild-type and NahG *N. benthamiana* plants. (**H**) SA levels in 21-day-old wild-type and NahG *N. benthamiana* plants upon whitefly attack. (**I**) Survival rate and fecundity of whiteflies on 21- and 35-day-old wild-type and NahG *N. benthamiana* plants. (**J**) Survival rate and fecundity of whiteflies on SA-treated (1 mM) 21-day-old wild-type *N. benthamiana* plants. (**K**) Survival rate and fecundity of whiteflies on SA-treated (1 mM) 35-day-old NahG *N. benthamiana* plants. Values are means ± SE, *n* = 40 for [(A) to (D)] and [(I) to (K)], *n* = 6 for [(E) to (G)], and *n* = 8 for (H). Student’s *t* test (two-tailed) was used for significant difference analysis in [(A) to (D)] and [(G) to (K)]. n.s., not significant. *P < 0.05, **P < 0.01. One-way analysis of variance (ANOVA) with Fisher’s least significant difference (LSD) test was used in [(E) and (F)]. Different lowercase letters indicate significant differences (*P* < 0.05).

To compare the defense levels of plants at different ages against phloem-feeding insects, we conducted bioassays of *B. tabaci* whiteflies, *Myzus persicae* aphids, and *Frankliniella occidentalis* western flower thrips on several Solanaceae species, including *N. benthamiana*, *Solanum lycopersicum* (tomato), and *Nicotiana tabacum* (tobacco). In *N. benthamiana*, both the survival rate and fecundity of whiteflies were significantly lower on adult plants (35 days old, with eight to nine true leaves) compared to juvenile plants (21 days old, with three to four true leaves) (Fig. 1, A and B). Similarly, whiteflies performed better on juvenile tobacco (21 days old, three to four true leaves) and tomato (21 days old, three to four true leaves) plants compared to adult plants (35 days old, six to seven true leaves for tobacco; eight to nine true leaves for tomato) (Fig. 1, A and B). In addition, western flower thrips showed higher survival rates, and aphids produced more offspring on juvenile plants compared to adult plants (Fig. 1, C and D). These results demonstrate ARR against phloem-feeding insects in Solanaceae plants.

### SA-dependent ARR against phloem-feeding insects

To explore the mechanism underlying ARR, we focused on *N. benthamiana*, which exhibited the most pronounced age-dependent insect resistance. We examined whether the defense phytohormones SA and JA were involved in ARR by comparing the basal levels of SA and JA in 21- and 35-day-old *N. benthamiana* plants. Meanwhile, to clarify potential age-related trends in phytohormone content, we expanded our analysis to include 28-day-old plants, representing an intermediate stage between the 21- and 35-day-old *N. benthamiana* plants. We observed a clear age-dependent increase in SA levels, with significantly higher levels in adult plants compared to juvenile plants (Fig. 1E). In contrast, JA levels showed no significant changes with age, and no differences were detected between juvenile and adult plants (Fig. 1F). This trend was also observed in tobacco (Fig. 1, E and F). In addition, the expression of *pathogenesis-related 1* (*NbPR1*), *pathogenesis-related 5* (*NbPR5*), and *enhanced disease susceptibility 1* (*NbEDS1*), three downstream markers of SA signaling (*34*–*36*), was significantly up-regulated with age, especially in adult plants, indicating enhanced SA signaling (fig. S1A).

To further investigate the role of SA in ARR, we used *N. benthamiana* transgenic plants expressing the *NahG* gene, which encodes *salicylate hydroxylase* from *Pseudomonas putida*, an enzyme that depletes SA accumulation (*37*). Unlike wild-type plants, NahG plants were unable to accumulate sufficient SA as they aged (Fig. 1G), and the mRNA levels of *NbPR1*, *NbPR5*, and *NbEDS1* were also significantly reduced in 35-day-old plants (fig. S1B). Moreover, whitefly infestation failed to induce SA accumulation in NahG plants (Fig. 1H). Bioassays revealed that whiteflies performed better on both juvenile and adult NahG plants compared to wild-type plants (Fig. 1I), indicating that both whitefly-inducible and growth-accumulated SA are critical for plant defense against whiteflies. Exogenous SA application further confirmed SA’s role in ARR. Treatment with SA increased *NbPR1*, *NbPR5*, and *NbEDS1* expression and enhanced whitefly defense in 21-day-old wild-type plants (Fig. 1J and fig. S1C), as well as in 35-day-old NahG plants (Fig. 1K). These results collectively confirm that ARR in *N. benthamiana* against whiteflies is SA dependent.

### SA-mediated ARR depends on *NbPAL6* expression

**Fig. 2.**
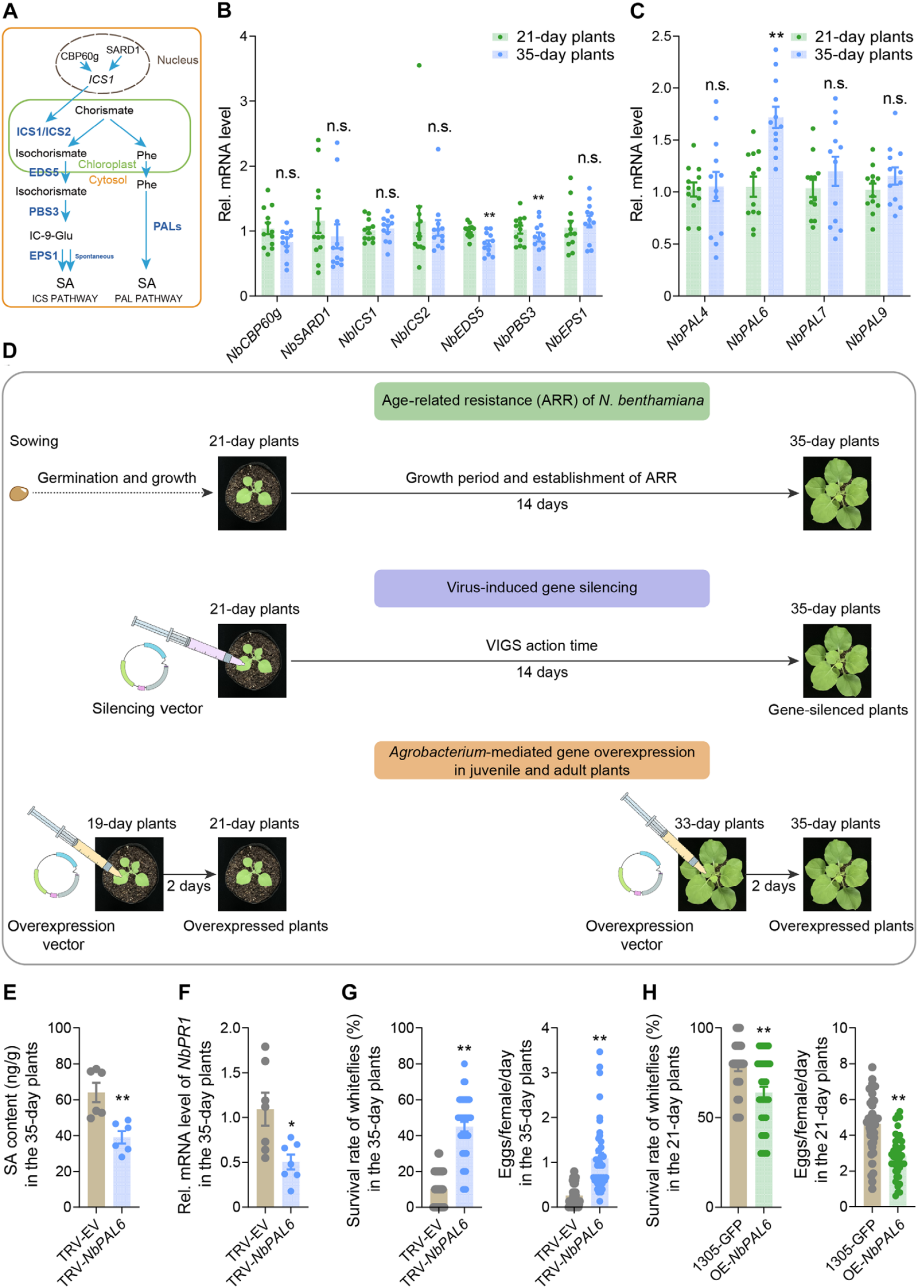
ARR depends on the expression of the SA synthetic gene *NbPAL6*. (**A**) SA synthesis pathways in plants. (**B**) Expression levels of genes in the ICS pathways in 21- and 35-day-old *N. benthamiana* plants. (**C**) Expression levels of *NbPAL* genes in 21- and 35-day-old *N. benthamiana* plants. (**D**) Experimental design of VIGS and gene overexpression in *N. benthamiana* plants. In the VIGS system, the pTRV2 vector is injected into 21-day-old plants, activating the silencing vector to inhibit target gene expression. Gene silencing typically became evident 14 days postinjection, aligning with the timeline for ARR establishment in *N. benthamiana*, making VIGS an effective tool for studying ARR-related gene functions. In comparison to control plants (receiving only the empty vector), 35-day-old plants subjected to gene silencing show reduced accumulation of target gene transcripts after reaching 14 days. In addition, *Agrobacterium*-mediated gene overexpression enhances target gene mRNA levels in both 21- and 35-day-old plants, allowing for gene function assessment across different developmental stages. (**E**) SA levels in *NbPAL6*-silenced 35-day-old *N. benthamiana* plants. (**F**) Expression level of *NbPR1* in *NbPAL6*-silenced 35-day-old *N. benthamiana* plants. (**G**) Survival rate and fecundity of whiteflies on *NbPAL6*-silenced 35-day-old *N. benthamiana* plants. (**H**) Survival rate and fecundity of whiteflies on *NbPAL6*-overexpressed 21-day-old *N. benthamiana* plants. Values are means ± SE, *n* = 12 for [(B) and (C)]; *n* = 7 for (F); *n* = 6 for (E); *n* = 40 for [(G) and (H)]. Student’s *t* test (two-tailed) was used for significant difference analysis. n.s., not significant. *P < 0.05, **P < 0.01.

In plants, SA is synthesized via two distinct pathways: the phenylalanine ammonia lyase (PAL) pathway and the isochorismate synthase (ICS) pathway (Fig. 2A) (*38*). To assess whether gene expression in these pathways changes with plant age, we performed reverse transcription quantitative polymerase chain reaction (RT-qPCR). The results showed no significant differences in the transcript levels of *NbICS1* and *NbICS2*, or the two TFs, *CaM-binding protein 60 g* (*CBP60g*) and *systemic acquired resistance deficient 1* (*SARD1*), which activate the ICS pathway (Fig. 2B). Similarly, components of the ICS pathway such as *enhanced disease susceptibility 5* (*EDS5*) and *AvrPphB susceptible 3* (*PBS3*) were down-regulated with plant age, while *enhanced pseudomonas susceptibility 1* (*EPS1*) remained unchanged (Fig. 2B). Given these findings, we shifted our focus to the PAL pathway.

A search of the *N. benthamiana* genome database (https://www.nbenth.com/) identified nine *PAL* genes (*NbPAL1*-*NbPAL9*), which we classified into four phylogenetic branches, likely representing alleles from the allotetraploid ancestry of *N. benthamiana* (fig. S2 and table S1). From these, we selected four *PAL* genes (*NbPAL4*, *NbPAL6*, *NbPAL7*, and *NbPAL9*) from the four branches, respectively, for further analysis. Expression profiling across developmental stages revealed that only *NbPAL6* showed increased transcription with plant age, while the other genes remained unchanged (Fig. 2C).

To determine whether *NbPAL6* is involved in SA-mediated ARR, we used virus-induced gene silencing (VIGS) and *Agrobacterium*-mediated overexpression to manipulate *NbPAL6* expression in *N. benthamiana* (*30*, *39*). The timing of these treatments was carefully designed to align with plant age requirements (Fig. 2D).

In the VIGS system, we injected the pTRV2 vector into 21-day-old plants, which led to effective gene silencing within 14 days (Fig. 2D). This period of transcriptional inhibition coincides with the establishment of ARR, making VIGS an ideal system for studying gene-related ARR (Fig. 2D). Compared to control plants (injected with an empty vector), 35-day-old VIGS-treated plants showed a significant reduction in target gene transcript accumulation after reaching 21 days of age. Subsequent validation experiments on the 35-day VIGS-treated plants provide valuable insights into the role of target genes in ARR establishment. Meanwhile, *Agrobacterium*-mediated overexpression can be used to enhance the mRNA level of target genes in both 21- and 35-day-old plants, enabling examination of gene function in plants of different ages (Fig. 2D). We injected 19-day-old plants with the pCAMBIA1305–green fluorescent protein (GFP) vector containing the target gene. Two days later, during the vector’s active period, elevated target gene mRNA levels were observed in the 21-day-old plant. By comparing overexpressed plants with control plants (injected with an empty vector), we could evaluate the effect of target genes on resistance in 21-day-old plants (Fig. 2D). Similarly, this method can also verify the effect of target gene overexpression in 35-day-old plants (Fig. 2D).

Our results showed that in VIGS-*NbPAL6* plants, only the expression of *NbPAL6* was significantly reduced, while the expression of other *NbPAL* genes remained unaffected, demonstrating the specificity of *NbPAL6* silencing in our VIGS system (fig. S3A). Furthermore, we found that 35-day-old VIGS-*NbPAL6* plants could not accumulate SA to the same levels as control (VIGS-EV) plants, nor could they up-regulate *NbPR1* expression (Fig. 2, E and F, and fig. S3A). Moreover, these plants exhibited reduced resistance to whiteflies (Fig. 2G). Conversely, overexpression of *NbPAL6* at 21 days of age significantly increased the SA and *NbPR1* levels and improved plant defense against whiteflies (Fig. 2H and fig. S3, B to D). These findings indicate that the differential expression of *NbPAL6* between juvenile and adult stages regulates SA level and SA-mediated ARR against whiteflies in *N. benthamiana*.

### Age-regulated NbMYB42 activates *NbPAL6* transcription

**Fig. 3.**
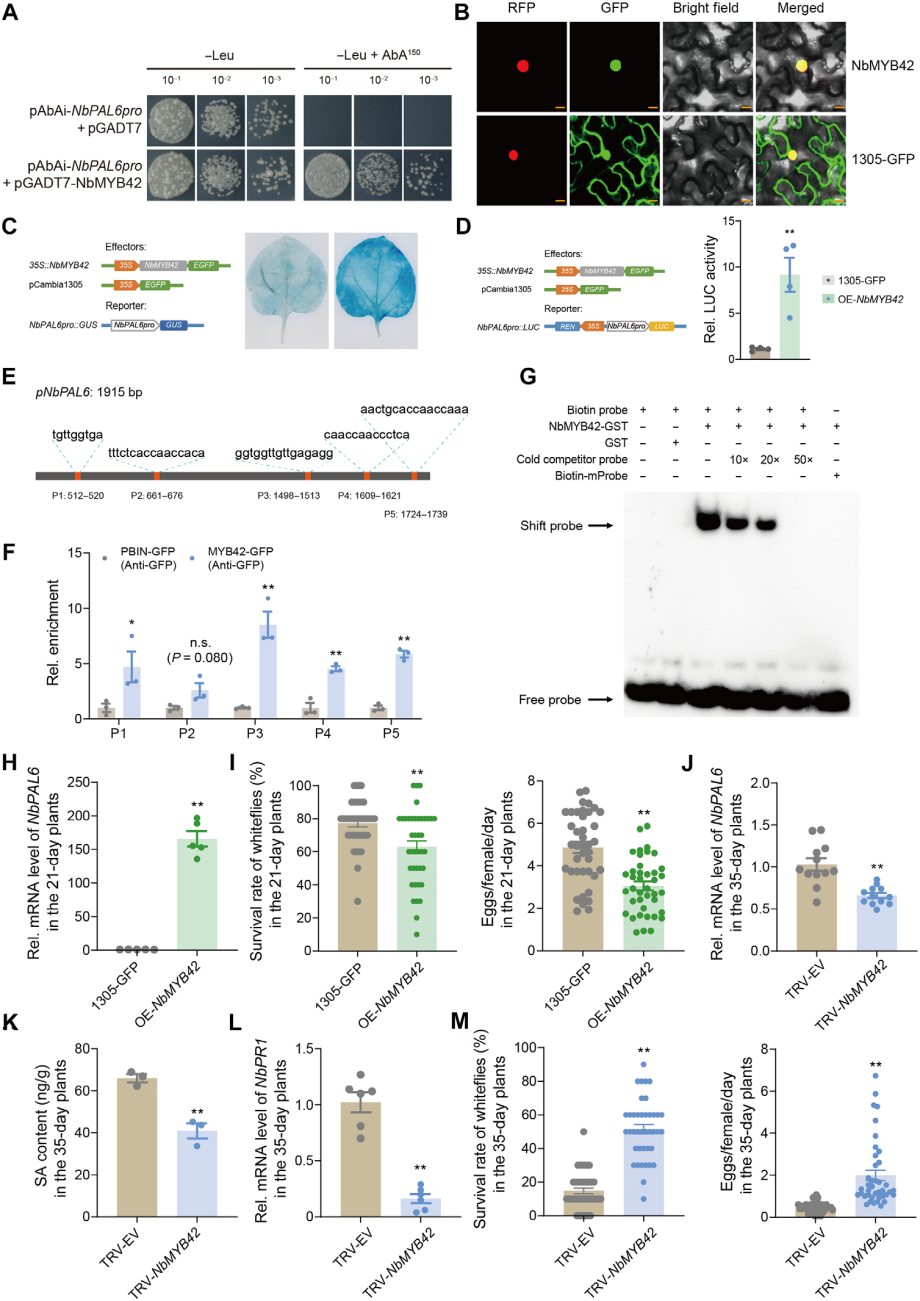
NbMYB42 directly activates *NbPAL6* transcription via promoter binding. (**A**) Y1H assay showing NbMYB42 interaction with *NbPAL6* promoter. pAbAi-*NbPAL6pro* and pGADT7-NbMYB42 cotransformed yeast grew on SD/-Leu medium with or without AbA (150 ng/ml). Empty pGADT7 served as control. (**B**) NbMYB42-GFP nuclear localization in *N. benthamiana* H2B-RFP line (control: *35S::GFP*). Scale bar, 20 μm. (**C** and **D**) Validation of NbMYB42 and *NbPAL6pro* interaction via GUS staining (C) and dual-luciferase assays (D). Diagrams show reporter/effector constructs (*35S*: promoter; *EGFP*/*GUS*/*LUC*/*REN*: reporters). REN activity normalized LUC signals. (**E**) *NbPAL6* promoter regions (P1 to P5) predicted as NbMYB42-binding sites. (**F**) ChIP-qPCR confirmed NbMYB42-GFP enrichment at *NbPAL6* promoter (anti-GFP antibody). Primers targeted regions in (E). (**G**) EMSA showing NbMYB42-GST binding to biotin-labeled *NbPAL6* promoter probe. Labeled probes were incubated with and without GST protein as negative controls, and 10-, 20-, and 50-fold excess unlabeled probes were used as cold competitors. Biotin-mProbe was the labeled probe containing mutations in the P3 element of the *NbPAL6* promoter. (**H**) Expression level of *NbPAL6* in *NbMYB42*-overexpressed 21-day-old *N. benthamiana* plants. (**I**) Survival rate and fecundity of whiteflies on *NbMYB42*-overexpressed 21-day-old *N. benthamiana* plants. (**J**) Expression level of *NbPAL6* in *NbMYB42*-silenced 35-day-old *N. benthamiana* plants. (**K**) SA levels in *NbMYB42*-silenced 35-day-old *N. benthamiana* plants. (**L**) Expression level of *NbPR1* in *NbMYB42*-silenced 35-day-old *N. benthamiana* plants. (**M**) Survival rate and fecundity of whiteflies on *NbMYB42*-silenced 35-day-old *N. benthamiana* plants. Values are means ± SE, *n* = 4 for [(D) and (F)]; *n* = 5 for (H); *n* = 40 for [(I) and (M)]; *n* = 12 for (J); *n* = 3 for (K); *n* = 6 for (L). Student’s *t* test (two-tailed) was used for significant difference analysis. n.s., not significant. *P < 0.05, **P < 0.01.

To identify the upstream TFs regulating *NbPAL6* expression, we screened the *NbPAL6* promoter (*NbPAL6pro*) using a yeast one-hybrid (Y1H) system with a cDNA library from *N. benthamiana*. NbMYB42, a TF belonging to the R2R3-MYB family, was identified. R2R3-MYB is known to regulate the phenylpropanoid metabolic pathway (*40*–*42*). Yeast transformants containing the plasmids pAbAi-*NbPAL6pro* and pGADT7-NbMYB42 were able to grow on synthetic defined (SD)/−Leu medium with AbA (150 ng/ml) within 3 days, while control transformants with pAbAi-*NbPAL6pro* and pGADT7 did not (Fig. 3A). This indicates that NbMYB42 can bind to the *NbPAL6* promoter. NbMYB42 was also found to localize in the nucleus (Fig. 3B), suggesting its role as a nuclear TF. To assess its role in activating *NbPAL6* expression in vivo, we performed the β-glucuronidase (GUS) staining assays. The results showed that *NbPAL6pro::GUS* coinfiltrated with *35S::NbMYB42* exhibited significantly higher GUS staining levels compared to the empty vector control in *N. benthamiana* leaves (Fig. 3C). Similarly, dual-luciferase reporter assays demonstrated increased luciferase activity when *NbPAL6pro::LUC* was coexpressed with *35S::NbMYB42* (Fig. 3D). These results confirmed that NbMYB42 activates *NbPAL6* promoter expression.

Next, we explored whether NbMYB42 directly binds to specific sites within the *NbPAL6pro*. Using the Jaspar database (https://jaspar.elixir.no/), we identified five potential MYB binding sites (P1 to P5) (Fig. 3E). To validate binding in vivo, we conducted chromatin immunoprecipitation (ChIP)–qPCR using plants overexpressing *NbMYB42* with GFP tag. We designed primers specific to the five binding sites to assess their interaction with NbMYB42 (table S2). The experiments were carried out using an anti-GFP antibody. Binding activity was observed at P1, P3, P4, and P5, but not P2, in the immunoprecipitated chromatin, indicating a significant accumulation of NbMYB42-GFP at these sites compared to the GFP control (Fig. 3F). Furthermore, electrophoretic mobility shift assay (EMSA) with recombinant NbMYB42–glutathione *S*-transferase (GST), purified from *Escherichia coli*, confirmed the binding activity in vitro. The MYB element site P3, which showed the strongest binding ability in the ChIP-qPCR test, was selected to prepare the biotin-labeled probe according to its sequence (table S2). We observed that the NbMYB42-GST protein was able to bind directly to the P3 probe, while the mutated probe eliminated NbMYB42-GST binding (Fig. 3G). In addition, an unlabeled P3 probe was prepared as the cold competitors. With the increase of competitor probes (10, 20, and 50 ×), the shifted band corresponding to the P3 probe and the NbMYB42 complex gradually disappeared (Fig. 3G), suggesting that the binding capacity was greatly reduced by the addition of unlabeled cold competition probe. These results collectively show that NbMYB42 specifically binds to the MYB cis element on the *NbPAL6* promoter.

We then studied whether NbMYB42 influences ARR against whiteflies by regulating NbPAL6-mediated SA accumulation. Overexpressing *NbMYB42* in 21-day-old *N. benthamiana* plants increased *NbPAL6* expression more than 150-fold compared to control plants (Fig. 3H and fig. S3E). The SA levels and SA-downstream *NbPR1* were also significantly up-regulated (fig. S3, F and G). This overexpression enhanced whitefly resistance in 21-day-old plants (Fig. 3I). Conversely, silencing *NbMYB42* in 35-day-old plants suppressed *NbPAL6* expression (Fig. 3J and fig. S3H), resulting in reduced SA and *NbPR1* levels (Fig. 3, K and L). Notably, the *NbMYB42*-silenced 35-day-old *N. benthamiana* plant showed significantly reduced whitefly defense compared to the control (Fig. 3M). These results indicate that NbMYB42 plays a crucial role in ARR in *N. benthamiana* by regulating *NbPAL6* expression and SA accumulation.

### Two NbARFs activate the expression of *NbMYB42*

**Fig. 4.**
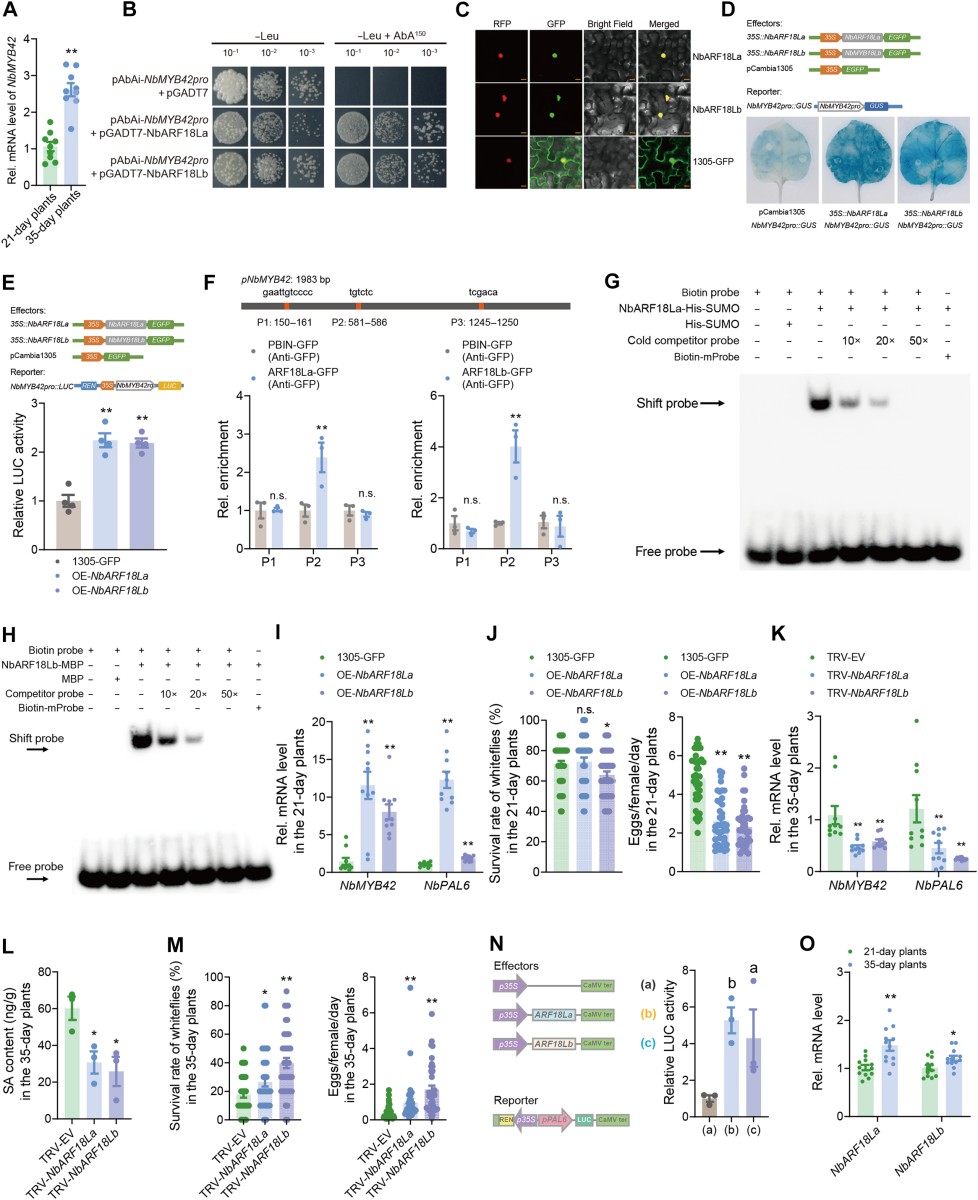
NbARF18La and NbARF18Lb activate *NbMYB42* expression. (**A**) Expression levels of *NbMYB42* in 21- and 35-day-old *N. benthamiana* plants. (**B**) Y1H assay showing NbARF18La/b interaction with *NbMYB42* promoter. Empty pGADT7 served as control. (**C**) NbARF18La/b-GFP nuclear localization in N. benthamiana H2B-RFP line (control: 35S::GFP). Scale bar, 20 μm. (**D** and **E**) Validation of NbARF18La/b and *NbMYB42pro* interaction via GUS staining (D) and dual-luciferase assays (E). Diagrams show reporter/effector constructs (*35S*: promoter; *EGFP*/*GUS*/*LUC*/*REN*: reporters). REN activity normalized LUC signals. (**F**) Top, NbMYB42 promoter regions (P1 to P3) predicted as NbARF-binding sites. Bottom, ChIP-qPCR confirmed NbARF18La/b-GFP enrichment at *NbMYB42* promoter (anti-GFP antibody). (**G** and **H**) EMSA showing NbARF18La-His-SUMO and NbARF18Lb-MBP binding to biotin-labeled *NbMYB42* promoter probe.10-, 20- and 50-fold excess unlabeled probes were used as cold competitor probes. (**I** and **K**) Expression levels of *NbMYB42* and *NbPAL6* in *NbARF18La/b*-overexpressed [21-day-old, (I)] and silenced [35-day-old, (K)] *N. benthamiana* plants. (**J** and **M**) Survival rate and fecundity of whiteflies on *NbMYB18a/b*-overexpressed [21-day-old, (J)] and silenced [35-day-old, (M)] *N. benthamiana* plants. (**L**) SA levels in *NbARF18La/b*-silenced 35-day-old *N. benthamiana* plants. (**N**) LUC activity driven by the *NbPAL6* promoter with different effectors. (**O**) Expression levels of *NbARF18La/b* in 21- and 35-day-old *N. benthamiana* plants. Values are means ± SE, *n* = 9 for (A); *n* = 4 for (E); *n* = 3 for [(F), (L), and (N)]; *n* = 10 for [(I) and (K)]; *n* = 40 for [(J) and (M)]; *n* = 12 for (O). Student’s *t* test (two-tailed) was used for significant difference analysis in [(A), (E), (F), (I) to (M), and (O)]. n.s., not significant. *P < 0.05, **P < 0.01. One-way ANOVA with Fisher’s LSD test was used in (N). Different lowercase letters indicate significant differences (*P* < 0.05).

We observed that *NbMYB42* expression was significantly higher in 35-day-old plants compared to 21-day-old plants (Fig. 4A), prompting further investigation into how *NbMYB42* expression is regulated during *N. benthamiana* development. Using a Y1H assay, we screened for TFs that regulate *NbMYB42* expression and identified two auxin response factors (ARFs) with distinct evolutionary relationships, which we named NbARF18La and NbARF18Lb (fig. S4A). Yeast strains harboring the pAbAi-*NbMYB42pro* and pGADT7-NbARF18La or pGADT7-NbARF18Lb constructs were able to grow on SD/−Leu medium supplemented with AbA (150 ng/ml), whereas yeast strains containing pAbAi-*NbMYB42pro* and a control pGADT7 plasmid could not (Fig. 4B), suggesting that both NbARF18La and NbARF18Lb bind to the *NbMYB42* promoter.

Phylogenetic analysis showed that NbARF18La and NbARF18Lb are closely related to ARF16 from *Arabidopsis thaliana* (fig. S4A). Amino acid sequence analysis revealed conserved B3-type DNA-binding domains in the N termini of both NbARF18La and NbARF18Lb and dimerization domains in their C termini (fig. S4B) (*43*–*46*). Subcellular localization analysis confirmed that both NbARFs are localized in the nucleus (Fig. 4C), indicating their function as nuclear TFs. To verify the activation of *NbMYB42* by these NbARFs, we conducted GUS staining and dual-luciferase reporter assays. Coexpression of *35S::NbARF18La* and *35S::NbARF18Lb* with the reporter *NbMYB42pro::GUS* in *N. benthamiana* leaves significantly increased *GUS* expression (Fig. 4D), and dual-luciferase reporter assay showed a significant up-regulation in LUC/REN ratio when *35S::NbARF18La* and *35S::NbARF18Lb* were coexpressed with the *NbMYB42pro::LUC* reporter (Fig. 4E). These results collectively demonstrate that NbARF18La and NbARF18Lb activate *NbMYB42* expression.

### DNA-binding specificity of NbARF18La and NbARF18Lb

To explore how the two NbARFs specifically bind to the *NbMYB42* promoter, we screened for potential auxin response elements (AuxRE) using the Jaspar database and identified three regions (P1, P2, and P3) in the promoter (Fig. 4F). We designed primers specific to these regions (table S2) and performed ChIP-qPCR using GFP-tagged NbARF18La/b and anti-GFP antibodies. The results revealed a significant enrichment of the P2 region, but not P1 and P3, in the immunoprecipitated chromatin, indicating that NbARF18La/b bind to the P2 AuxRE region (TGTCTC) in the *NbMYB42* promoter (Fig. 4F).

To confirm the binding specificity, we conducted an EMSA. The recombinant NbARF18La-His-SUMO fusion protein bound to the P2 probe of the *NbMYB42* promoter, while no binding occurred with a mutant probe (TGTCTC to AAAAAA) (Fig. 4G). In addition, competition assays using unlabeled probes verified the specificity of binding (Fig. 4G). Similarly, the NbARF18Lb–maltose-binding protein (MBP) fusion protein bound to the same promoter region (P2), but not to the mutant probe, and competition with unlabeled probes also reduced the binding strength (Fig. 4H). Together, these results indicate that NbARF18La and NbARF18Lb specifically bind to the same AuxRE site in the *NbMYB42* promoter.

### NbARF18La and NbARF18Lb interaction and self-association

Previous studies have shown that ARF TFs can form complexes through self-interactions or by interacting with other TFs (*43*–*45*, *47*). To explore potential interactions between NbARF18La and NbARF18Lb or self-interactions of either of the TFs, we performed yeast two-hybrid (Y2H) assays. Yeast cells expressing NbARF18La-AD/NbARF18Lb-BD grew on selective media (SD/−Leu–Trp–His–Ade), indicating that NbARF18La and NbARF18Lb can interact with each other (fig. S5A). In addition, NbARF18La was able to interact with itself in Y2H assays (fig. S5A).

We then confirmed these interactions in vivo using a bimolecular fluorescence complementation (BiFC) assay. GFP fluorescence was detected in *N. benthamiana* leaves coexpressing *NbARF18La* and *NbARF18Lb* or *NbARF18La* alone (fig. S5B). In vitro pull-down assays further validated these interactions, as NbARF18La-His exhibited an affinity for NbARF18Lb-MBP, but no interaction was observed in the negative control (fig. S5C). These results demonstrate that NbARF18La and NbARF18Lb physically interact and NbARF18La can self-interact, suggesting that these interactions may be important for cooperatively regulating *NbMYB42* expression.

### NbARFs regulate plant whitefly resistance in an age-related manner

Next, we examined the role of NbARF18La and NbARF18Lb in ARR to whiteflies in *N. benthamiana*. Overexpression of both *NbARFs* significantly up-regulated expression of *NbMYB42*, *NbPAL6*, and the downstream *NbPR1* gene in 21-days-old plants (Fig. 4I and fig. S6, A to C), which led to enhanced resistance to whiteflies (Fig. 4J). In contrast, silencing *NbARF* genes via VIGS in 35-day-old *N. benthamiana* plants resulted in substantially reduced expression of *NbMYB42* and *NbPAL6* (Fig. 4K and fig. S6, D and E), as well as significantly decreased endogenous SA levels and reduced *NbPR1* expression (Fig. 4L and fig. S6F). Correspondingly, silencing *NbARFs* impaired the defense response against whiteflies in adult plants (Fig. 4M).

To further investigate how NbARFs regulate *NbPAL6* expression, we coexpressed *NbARF18La/b* with an LUC reporter fused to the *NbPAL6* promoter, which significantly enhanced LUC activity (Fig. 4N), indicating that NbARF18La/b regulate *NbPAL6* by modulating the expression of *NbMYB42*. Furthermore, expression of *NbARF18La/b* was significantly higher in 35-day-old plants than in 21-day-old plants (Fig. 4O), indicating that the NbARF18La/b-NbMYB42-NbPAL6 module is regulated in an age-dependent manner.

### Auxin suppresses juvenile plant defense by repressing SA

**Fig. 5.**
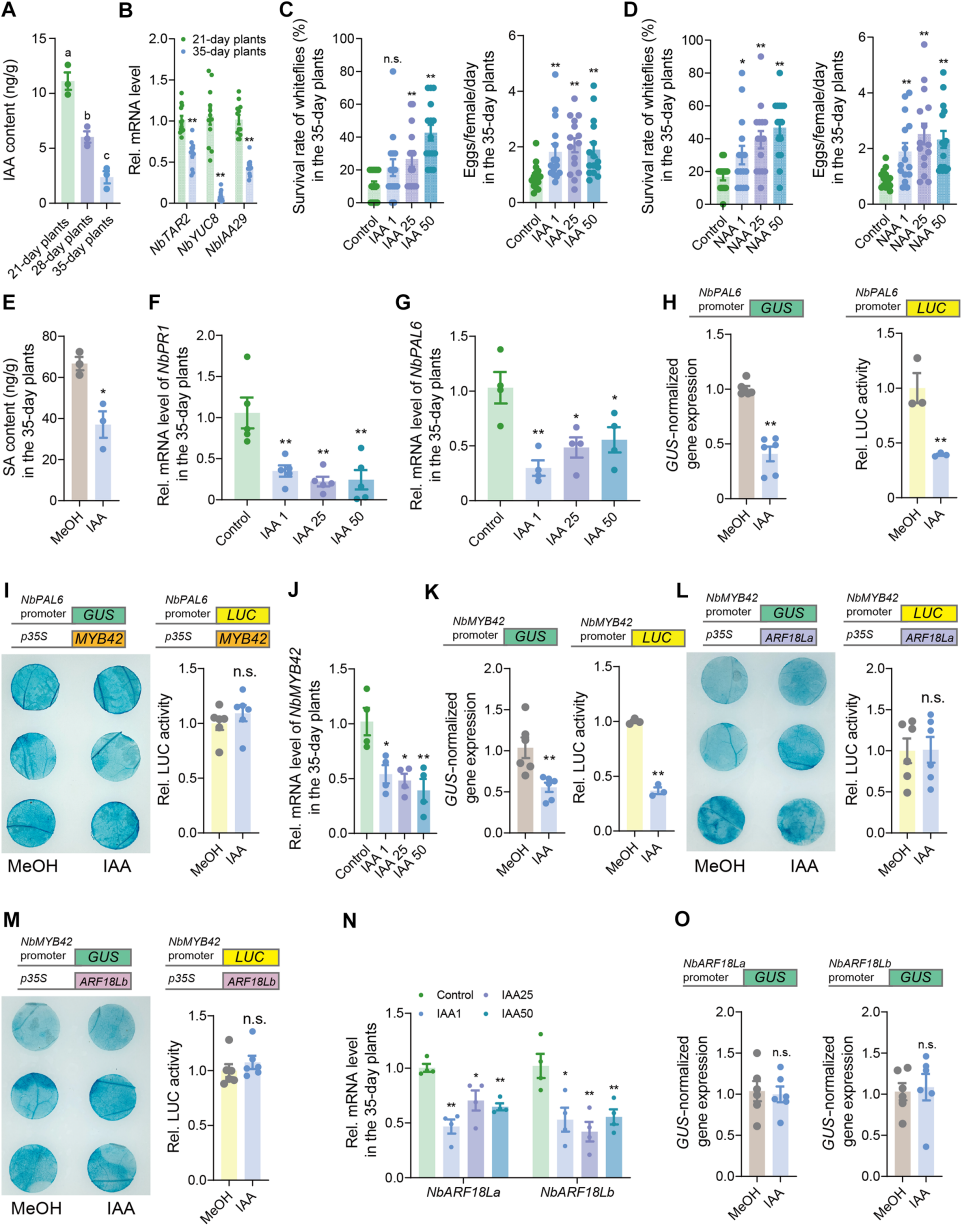
Auxin suppresses NbARF18La/b-NbMYB42–mediated SA accumulation. (**A**) IAA levels in 21-, 28-, and 35-day-old *N. benthamiana* plants. (**B**) Expression level of *NbTAR2*, *NbYUC8*, and *NbIAA29* in 21- and 35-day-old plants. (**C** and **D**) Survival rate and fecundity of whiteflies on IAA/NAA-treated (1, 25, and 50 μM) 35-day-old plants. (**E**) SA levels in IAA-treated (25 μM) 35-day-old plants. (**F** and **G**) Expression level of *NbPR1* (F) and *NbPAL6* (G) in IAA-treated (1, 25, and 50 μM) 35-day-old plants. (**H**) *GUS* expression in mock- and IAA-treated (25 μM) *pNbPAL6::GUS* plants. (**I**) GUS / LUC activity coexpressed with *NbMYB42* in mock- or IAA-treated (25 μM) *pNbPAL6::GUS* plants. (**J**) Expression level of *NbMYB42* in IAA-treated (1, 25, and 50 μM) 35-day-old plants. (**K**) Top, schematic of the *GUS* reporter genes. Bottom, *GUS* expression in mock- and IAA-treated (25 μM) *pNbMYB42::GUS* plants. (**L** and **M**) GUS/LUC activity coexpressed with *NbARF18La* (L) or *NbARF18Lb* (M) in mock- and IAA-treated (25 μM) *pNbMYB42::GUS/LUC* plants. (**N**) Expression level of *NbARF18La/b* in IAA-treated (1, 25, and 50 μM) 35-day-old plants. (**O**) *GUS* expression in mock- and IAA-treated (25 μM) *pNbARF18La/b::GUS* plants. Values are means ± SE, *n* = 3 for [(A), (E), (H)] (LUC activity), and (K) (LUC activity); *n* = 12 for (B); *n* = 15 for [(C) and (D)]; *n* = 5 for (F); *n* = 4 for [(G), (J), and (N)]; *n* = 6 for [(H), (K), and (O)] (*GUS* expression); *n* = 6 for [(I), (L), and (M)] (LUC activity). Statistical analysis: Student’s *t* test (two-tailed) was used for significant difference analysis in [(B) to (O)]. n.s., not significant. *P < 0.05, **P < 0.01. One-way ANOVA with Fisher’s LSD test was used in (A). Different lowercase letters indicate significant differences (*P* < 0.05).

Given the findings above, we questioned why SA-mediated defense is not fully established during the juvenile stage of plants. Since defense-related ARF TFs are key components downstream of the auxin response, we postulated that auxin is involved in this process. To test this hypothesis, we first measured the levels of indole-3-acetic acid (IAA) in 21-, 28-, and 35-day-old plants to examine age-related trends in IAA accumulation. We found that IAA levels decreased with age, with significantly higher levels in juvenile plants compared to adult plants (Fig. 5A). In parallel, we observed elevated expression levels of the auxin synthesis genes, *TRYPTOPHAN AMINOTRANSFERASE RELATED 2* (*NbTAR2*) and *YUCCA 8* (*NbYUC8*), as well as the auxin-responsive gene *NbIAA29*, in juvenile plants (Fig. 5B).

To assess the effect of auxin on plant defense, we treated 35-day-old plants, which are known for their high resistance to whiteflies, with natural IAA and the auxin-like plant growth regulator, 1-naphthalene acetic acid (NAA). Bioassays revealed that the whiteflies performed better on IAA- and NAA-treated plants, indicating that auxin negatively affects plant defense (Fig. 5, C and D). Furthermore, IAA treatment significantly suppressed both the SA levels (Fig. 5E) and the expression of the SA-responsive gene *NbPR1* (Fig. 5F) in the 35-day-old plants.

To further clarify the role of auxin in repressing SA-based resistance, we treated 21-day-old plants with two auxin biosynthesis inhibitors: _L_-kynurenine (_L_-Kyn), a competitive substrate inhibitor, and Yucasin [5-(4-chlorophenyl)-4H-1,2,4-triazole-3-thiol], an effective IAA biosynthesis inhibitor (*48*). Plants treated with these auxin inhibitors showed increased resistance to whiteflies (fig. S7, A and B) as well as higher expression levels of *NbPR1* (fig. S7C). These results confirm that auxin suppresses SA biosynthesis and downstream signaling pathways in juvenile *N. benthamiana* plants, thereby repressing SA-mediated defense against whiteflies.

### Auxin suppresses *NbPAL6* and *NbMYB42* expression via *NbARF*

To explore how auxin suppresses SA accumulation, we focused on the age-regulated SA synthesis gene *NbPAL6*. Auxin treatment significantly reduced *NbPAL6* expression in 35-day-old plants (Fig. 5G), whereas treatment with an auxin inhibitor enhanced *NbPAL6* expression in 21-day-old plants (fig. S7D). To investigate the underlying mechanism, we used *NbPAL6* promoter–driven *GUS* and *LUC* reporters, which showed decreased transcript levels upon auxin treatment, indicating that auxin suppresses *NbPAL6* expression through transcriptional repression (Fig. 5H).

We hypothesized that auxin may inhibit *NbPAL6* transcription by suppressing the activity of NbMYB42. However, *NbPAL6* promoter–linked *GUS* and *LUC* reporter assays in IAA-treated plants revealed no significant impact on reporter expression (Fig. 5I), suggesting that auxin does not affect the transactivation ability of NbMYB42.

Next, we assessed whether auxin affects *NbMYB42* mRNA levels. RT-qPCR revealed a significant reduction in *NbMYB42* mRNA levels in auxin-treated 35-day-old plants (Fig. 5J), whereas auxin inhibitor treatment enhanced *NbMYB42* expression in 21-day-old plants (fig. S7E). A reporter-GUS assay further confirmed that auxin represses *NbMYB42* transcription (Fig. 5K). These results indicate that auxin represses *NbMYB42* transcription, thereby regulating downstream *NbPAL6* expression.

To understand how auxin represses *NbMYB42* transcription, we examined upstream TFs, NbARF18La and NbARF18Lb. Unexpectedly, auxin treatment did not affect the activation of *NbMYB42* by NbARF18La/b (Fig. 5, L and M). However, auxin significantly decreased the transcript levels of *NbARF18La/b* in 35-day-old plants (Fig. 5N), with auxin inhibitors increasing their expression in 21-day-old plants (fig. S7F). These results indicate that auxin down-regulates NbARF18La/b, which in turn suppresses *NbMYB42*. Further, we examined whether auxin repressed *NbARFs* through transcriptional inhibition. Using promoter-*GUS* reporters, we found that auxin did not reduce *NbARF18La/b* mRNA levels through transcriptional repression (Fig. 5O). Therefore, it appears that auxin suppresses *NbARF* mRNA levels through a posttranscriptional regulatory mechanism.

### Auxin-inducible miRNA *Nb*miR160c targets *NbARF* mRNA

**Fig. 6.**
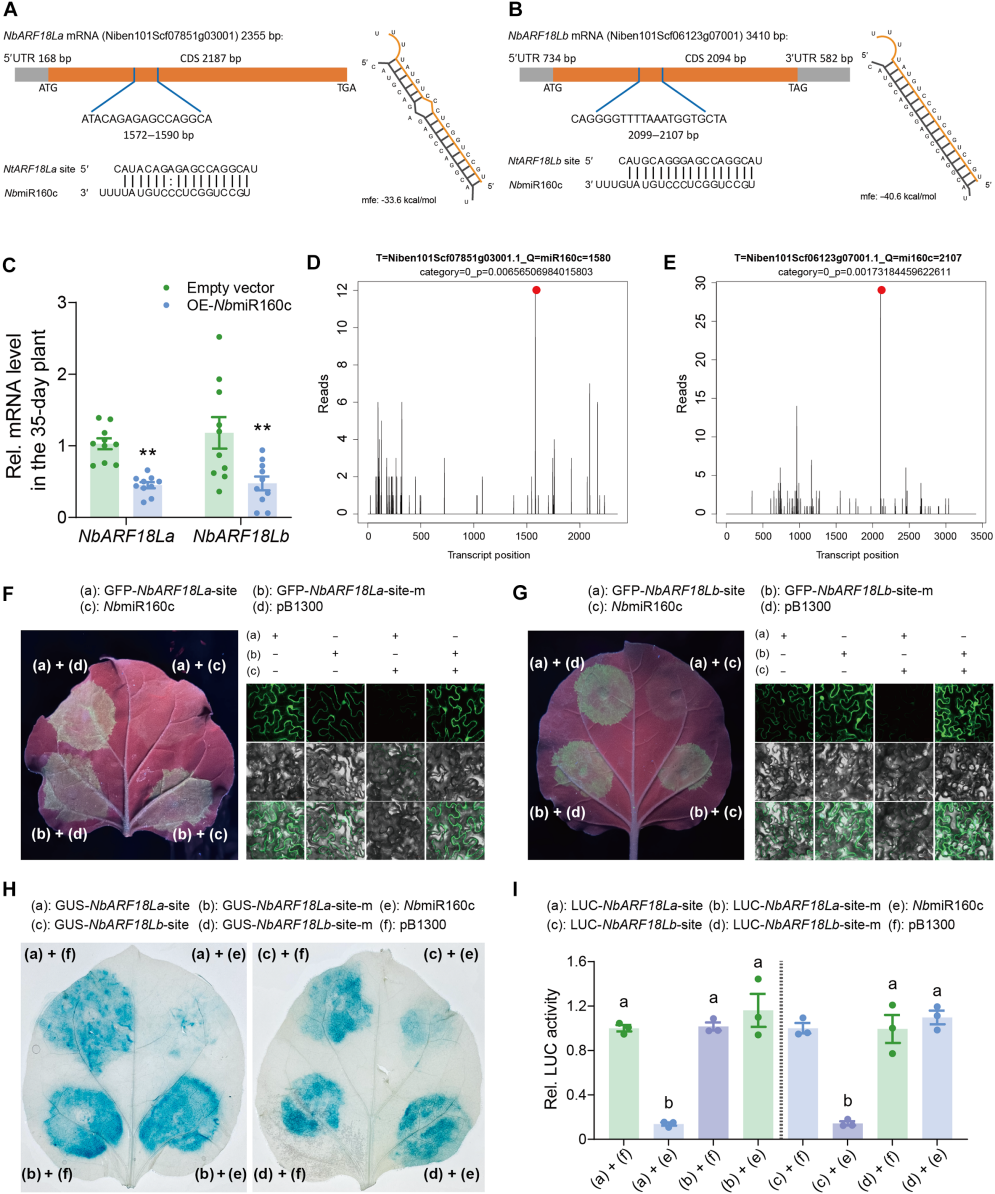
Auxin-induced microRNA160c targets *NbARF18La/b* transcripts. (**A**) *Nb*miR160c targets *N. benthamiana NbARF18La* mRNA. CDS, coding sequence; UTR, untranslated regions; ATG, initiation codon; TAG and TGA, termination codon. (**B**) *Nb*miR160c targets *N. benthamiana NbARF18Lb* mRNA. (**C**) Expression level of *NbARF18La/b* in *Nb*miR160c-expressing 35-day-old *N. benthamiana* plants. (**D**) Target plots (t-plots) of *Nb*miR160c and its target *NbARF18La* mRNA, validated via degradome sequencing. The red dot indicates the cleavage site of the *NbARF18La* transcript at 1572 bp, as analyzed by GSTAr (1.0) cleavage site prediction software. (**E**) T-plots of *Nb*miR160c and its target *NbARF18Lb* mRNA, showing cleavage at 2099 bp. (**F** and **G**) Camera (left) and confocal (right) images of GFP-*NbARF18La/b* target site and GFP-*NbARF18La/b* mutant site expression in *N. benthamiana* when *Nb*miR160c was overexpressed. Top, GFP; middle, bright-field; bottom, GFP/bright-field overlay. Scale bar, 40 μm. (**H**) GUS phenotype observed by histochemical staining showing GFP-*NbARF18La/b* target site and *GFP-NbARF18La/b* mutant site expression in *N. benthamiana* with overexpressed *Nb*miR160c. (**I**) LUC activity displaying LUC-*NbARF18La/b* target site and LUC-*NbARF18La/b* mutant site expression. Values are means ± SE, *n* = 10 for (C); *n* = 3 for (I). Student’s *t* test (two-tailed) was used for significant difference analysis in (C). **P < 0.01. One-way ANOVA with Fisher’s LSD test was used in (I). Different lowercase letters indicate significant differences (*P* < 0.05).

We explored the mechanisms by which auxin suppresses the transcript levels of *NbARF18La* and *NbARF18Lb* via posttranscriptional regulation. Previous studies have shown that plant miRNAs can negatively regulate the transcripts of *ARF* genes (*49*, *50*) and that auxin treatment can enhance miRNA accumulation in plants (*51*). Therefore, we hypothesized that auxin-inducible, age-related miRNAs may be responsible for the posttranscriptional regulation of these *ARF* genes. To test this hypothesis, we analyzed miRNA sequencing data from 21-day-old *N. benthamiana* plants and, through target prediction analysis, identified a miRNA, *Nb*miR160c, which potentially targets the transcripts of *NbARF18La* and *NbARF18Lb*. Target site analysis revealed that *Nb*miR160c specifically binds to the coding regions of *NbARF18La* [1572 to 1590 base pairs (bp)] and *NbARF18Lb* (2099 to 2107 bp) (Fig. 6, A and B). To validate this interaction, we artificially overexpressed *Nb*miR160c in 35-day-old plants. This overexpression significantly reduced the mRNA levels of *NbARF18La* and *NbARF18Lb* (Fig. 6C and fig. S8, A and B), suggesting direct regulation of these transcripts by *Nb*miR160c.

Next, to pinpoint the exact cleavage sites of *NbARF18La* and *NbARF18Lb* by *Nb*miR160c in juvenile plants, we conducted degradome sequencing on samples from 21-day-old plants. The analysis confirmed that *Nb*miR160c cleaves *NbARF18La* and *NbARF18Lb* transcripts at the predicted target sites, as shown by the accumulation of cleavage products at these regions (Fig. 6, D and E). The red dots in Fig. 6 (D and E) highlight the cleavage sites, aligning with the *Nb*miR160c target predictions.

To further confirm the regulatory role of *Nb*miR160c, we cloned the target sites of *NbARF18La* and *NbARF18Lb* and fused them with a GFP tag. When these constructs were transiently expressed in *N. benthamiana* leaves, coexpression with *Nb*miR160c resulted in the suppression of GFP fluorescence (Fig. 6, F and G, and fig. S8, C and D). However, when the *Nb*miR160c target sites were mutated, *GFP* expression was unaffected, even in the presence of *Nb*miR160c (Fig. 6, F and G, and fig. S8, C and D). In addition, in coexpression assays with GUS-tagged target sites, *Nb*miR160c suppressed the expression of wild-type GUS-tagged *NbARF18La* and *NbARF18Lb* target sites, while the mutant target sites were unaffected (Fig. 6H). We observed similar results using LUC-tagged reporter constructs, where *Nb*miR160c suppressed the activity of the LUC reporter fused to the wild-type target sites of *NbARF18La* and *NbARF18Lb*, but not the mutant constructs (Fig. 6I). Collectively, these results confirm that *Nb*miR160c directly targets the coding regions of *NbARF18La* and *NbARF18Lb*, leading to their posttranscriptional repression. This auxin-inducible miRNA thereby plays a crucial role in modulating *ARF* expression in *N. benthamiana*, particularly during the juvenile stage.

### *Nb*miR160c regulates the expression of *NbMYB42* and *NbPAL6*

**Fig. 7.**
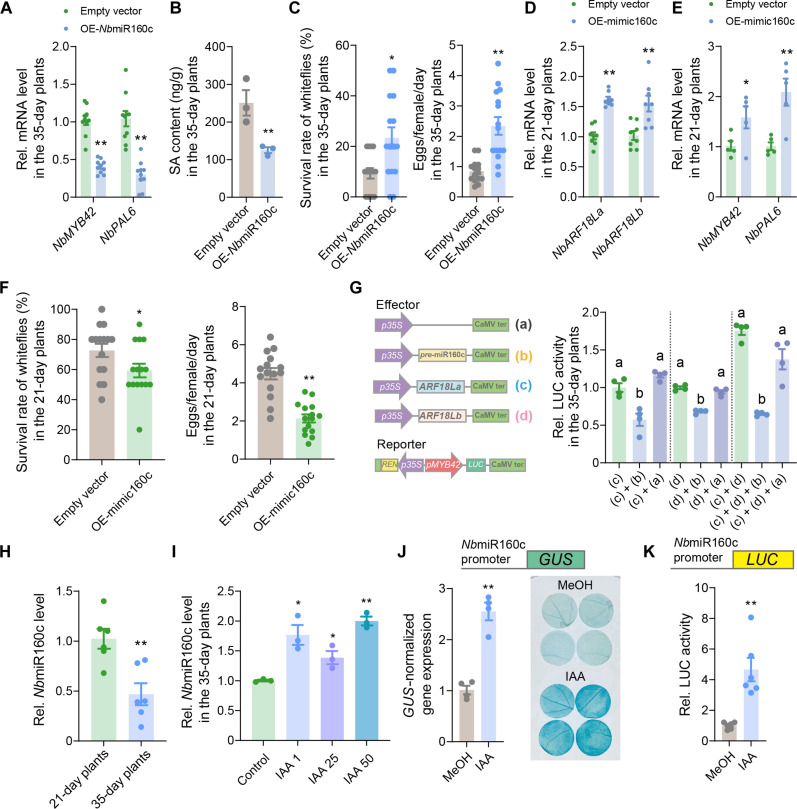
Auxin-induced microRNA160c negatively regulates plant defense. (**A**) Expression level of *NbMYB42* and *NbPAL6* in *Nb*miR160c-expressing 35-day-old *N. benthamiana* plants. (**B**) SA levels in *Nb*miR160c-expressing 35-day-old *N. benthamiana* plants. (**C**) Survival rate and fecundity of whiteflies on *Nb*miR160c-expressing 35-day-old *N. benthamiana* plants. (**D**) Expression level of *NbARF18La/b* in mimic160c-expressing 21-day-old *N. benthamiana* plants. (**E**) Expression level of *NbMYB42* and *NbPAL6* in mimic160c-expressing 21-day-old *N. benthamiana* plants. (**F**) Survival rate and fecundity of whiteflies on the mimic160c-expressing 21-day-old *N. benthamiana* plants. (**G**) Left, schematic diagram showing effector and reporter vectors used in transient transcriptional activity assays in *N. benthamiana* leaves. Right, the LUC activity driven by the *NbMYB42* promoter when coexpressed with different effectors. (**H**) Expression level of *Nb*miR160c in 21- and 35-day-old *N. benthamiana* plants. (**I**) *Nb*miR160c levels in IAA-treated (1, 25, and 50 μM) 35-day-old plants. (**J**) Top, schematic of the *GUS* reporter gene; bottom, *GUS* reporter gene expression and activity in mock- and IAA-treated (25 μM) *pNb*miR160c*::GUS N. benthamiana* plants. (**K**) Top, schematic of the *LUC* reporter gene; bottom, *LUC* reporter gene expression and activity in mock- and IAA-treated (25 μM) *pNb*miR160c*::LUC N. benthamiana* plants. Values are means ± SE; *n* = 10 for (A); *n* = 3 for [(B) and (I)]; *n* = 15 for [(C) and (F)]; *n* = 8 for (D); *n* = 5 for (E); *n* = 6 for [(H) and (K)]; *n* = 4 for [(G) and (J)]. Student’s *t* test (two-tailed) was used for significant difference analysis in [(A) to (F) and (H) to (K)]. *P < 0.05, **P < 0.01. One-way ANOVA with Fisher’s LSD test was used in (G). Different lowercase letters indicate significant differences (*P* < 0.05).

To explore the role of *Nb*miR160c in regulating the NbARF-NbMYB42 module–mediated SA defense against whiteflies, we first overexpressed *Nb*miR160c in 35-day-old plants. The overexpression resulted in a significant reduction in the mRNA levels of *NbMYB42* and *NbPAL6* (Fig. 7A), as well as in SA content and downstream *NbPR1* expression (Fig. 7B and fig. S8E). Notably, the *Nb*miR160c-expressing 35-day-old plants exhibited reduced defense against whiteflies (Fig. 7C), indicating that *Nb*miR160c negatively regulates plant anti-insect defense.

To validate the role of *Nb*miR160c in repressing *NbARF18La/b* and downstream SA regulatory pathways in juvenile plants, we overexpressed an artificial target mimic of *Nb*miR160c (mimic160c) in 21-day-old plants to inhibit its function (fig. S8F). As expected, *NbARF18La* and *NbARF18Lb* transcript levels were significantly up-regulated in the treated plants (Fig. 7D). Moreover, the *NbMYB42* and *NbPAL6* mRNA levels (Fig. 7E), SA levels (fig. S8G), and *NbPR1* expression were also significantly elevated in the mimic160c-overexpressing plants (fig. S8H). Correspondingly, bioassays showed that whiteflies performed poorly on the mimic160c-overexpressing plants, indicating enhanced defense (Fig. 7F).

We then examined the effect of *Nb*miR160c on NbARF-promoted *NbMYB42* transcription in plants at juvenile and adult stages. In 35-day-old plants, overexpression of *Nb*miR160c repressed the activity of the *NbMYB42pro*-*LUC* reporter (fig. S9A), whereas overexpression of mimic160c enhanced *NbMYB42pro*-*LUC* reporter expression in 21-day-old plants (fig. S9B). When *Nb*miR160c was coexpressed with *NbARF18La*, *NbARF18Lb*, or both, *NbMYB42pro* reporter expression was significantly inhibited in 35-day-old plants (Fig. 7G). These results demonstrate that *Nb*miR160c represses NbARF-activated *NbMYB42* transcription. Similarly, we confirmed that *Nb*miR160c is involved in similarly repressing *NbPAL6* transcription (fig. S9, C to E). Collectively, these findings show that *Nb*miR160c negatively regulates *NbARF18La* and *NbARF18Lb*, thereby affecting the transcription of *NbMYB42* and its downstream target *NbPAL6*.

### Auxin promotes the *Nb*miR160c level by enhancing its expression

Next, we investigated the relationship between the *Nb*miR160c expression, plant age, and phytohormones. We observed that *Nb*miR160c levels were significantly higher in 21-day-old plants compared to 35-day-old plants (Fig. 7H). Given that auxin content is typically higher in juvenile plants, we speculated that auxin might promote *Nb*miR160c expression. To test this hypothesis, we treated 35-day-old plants, which had naturally lower *Nb*miR160c levels, with IAA. The treatment significantly increased *Nb*miR160c levels (Fig. 7I). In addition, we cloned the promoter of the *Nb*miR160c precursor from the *N. benthamiana* genome and fused it to *GUS* and *LUC* reporters. Upon IAA treatment, we observed enhanced reporter expression (Fig. 7, J and K), confirming that auxin activates *Nb*miR160c transcription. These results indicate that auxin promotes *Nb*miR160c expression, thereby repressing the NbARF-NbMYB42-NbPAL6 module, which mediates whitefly defense in juvenile *N. benthamiana* plants.

### Excessive SA affects early plant growth by antagonizing auxin

**Fig. 8.**
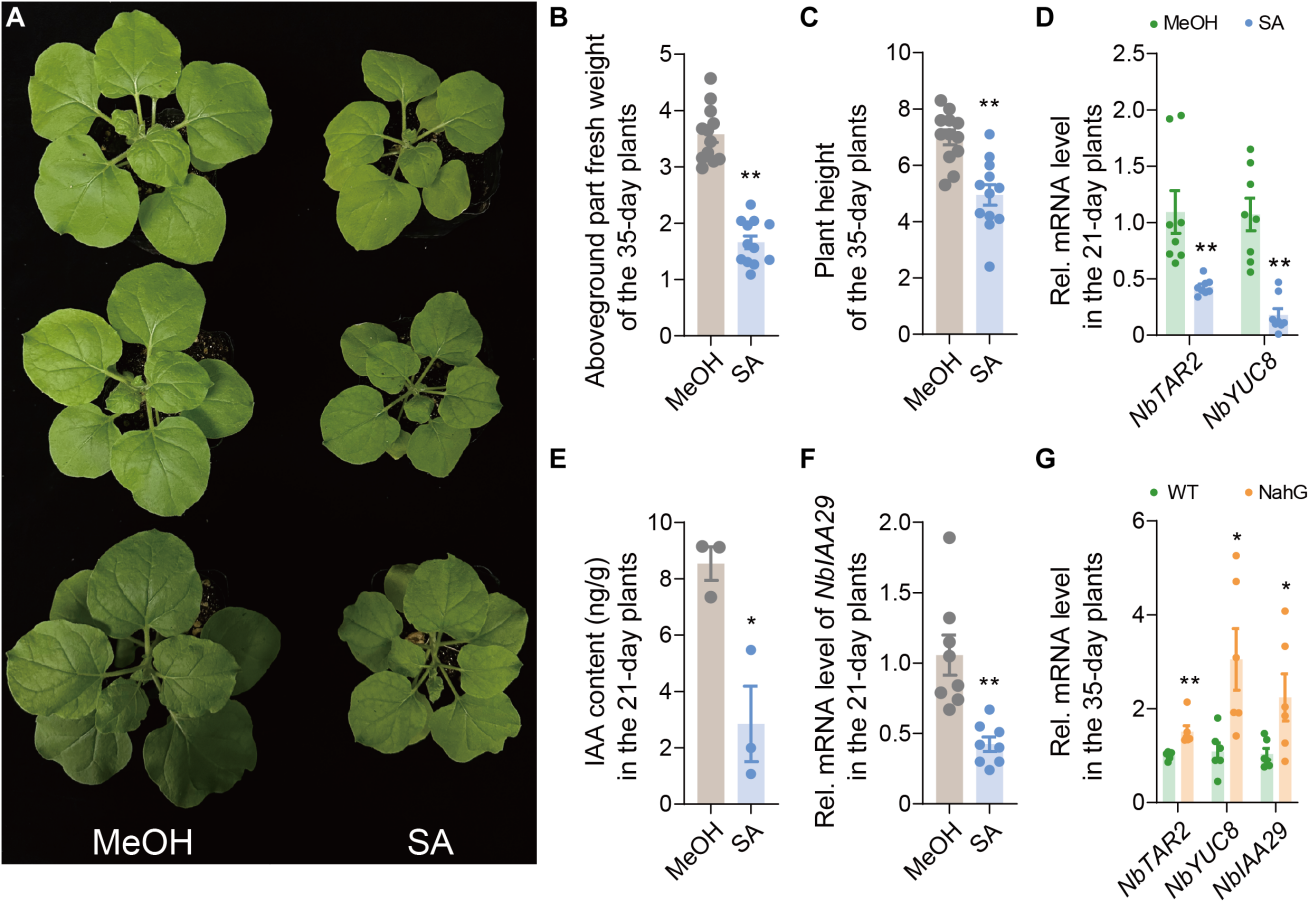
Excessive SA affects early plant growth by antagonizing auxin. (**A**) Photographs of 35-day-old *N. benthamiana* plants treated with SA starting at 21 days old. (**B** and **C**) Aboveground fresh weight (B) and plant height (C) of SA-treated (1 mM) *N. benthamiana* plants. (**D**) Expression level of auxin synthesis genes *NbTAR2* and *NbYUC8* in 21-day-old *N. benthamiana* plants treated with 1 mM SA. (**E**) Auxin level in 21-day-old *N. benthamiana* plants after successive SA treatment (1 mM). (**F**) Expression level of auxin downstream gene *NbIAA29* in SA-treated (1 mM) 21-day-old *N. benthamiana* plants. (**G**) Expression level of auxin pathway genes in 35-day-old wild-type and NahG *N. benthamiana* plants. Values are means ± SE, *n* = 12 for [(B) and (C)]; *n* = 8 for [(D) and (F)]; *n* = 3 for (E); *n* = 6 for (G). Student’s *t* test (two-tailed) was used for significant difference analysis. *P < 0.05, **P < 0.01.

We further examined the physiological implications of auxin-mediated suppression of SA in juvenile plants. To assess the impact of excessive SA on early plant growth, we treated 21-day-old plants with exogenous SA. These treated plants showed stunted growth (Fig. 8A) and, by the time they reached 35 days of age, had significantly reduced weight and height compared to untreated plants (Fig. 8, B and C). These results, along with our earlier findings, suggest that while excessive SA enhances whitefly defense in juvenile plants, it also negatively affect plant early growth.

Since auxin and SA pathways are known to antagonize each other, we examined whether SA inhibited early growth by interfering with the auxin pathway. Our results show that SA treatment significantly reduced the expression of the auxin synthesis genes *NbTAR2* and *NbYUC8* (Fig. 8D), lowered IAA levels (Fig. 8E), and decreased downstream *NbIAA29* expression (Fig. 8F) in juvenile plants. Conversely, the NahG plants, which have reduced SA levels, showed enhanced expression of auxin synthesis genes compared to wild-type plants at 35 days (Fig. 8G). These results demonstrate that excessive SA in the early stages of plant development inhibits auxin accumulation, thereby hindering normal growth.

## DISCUSSION

In nature, plants exhibit enhanced defense against herbivorous insects as they age, with juvenile plants experiencing more frequent attacks than their adult counterparts. This observation has led to the development of the concepts of ARR and PVH; however, the precise mechanisms underlying these phenomena remain largely unknown. In this study, we elucidate the fine regulation of SA-mediated ARR against phloem-feeding whiteflies in plants (Fig. 1). We highlight the importance of the PAL synthesis pathway in age-related intrinsic SA accumulation, identifying the key synthetic gene *NbPAL6* in *N. benthamiana* (Fig. 2). In addition, we identify the age-related upstream TFs NbMYB42 and NbARF18La/b that are involved in SA regulation (Figs. 3 and 4).

**Fig. 9.**
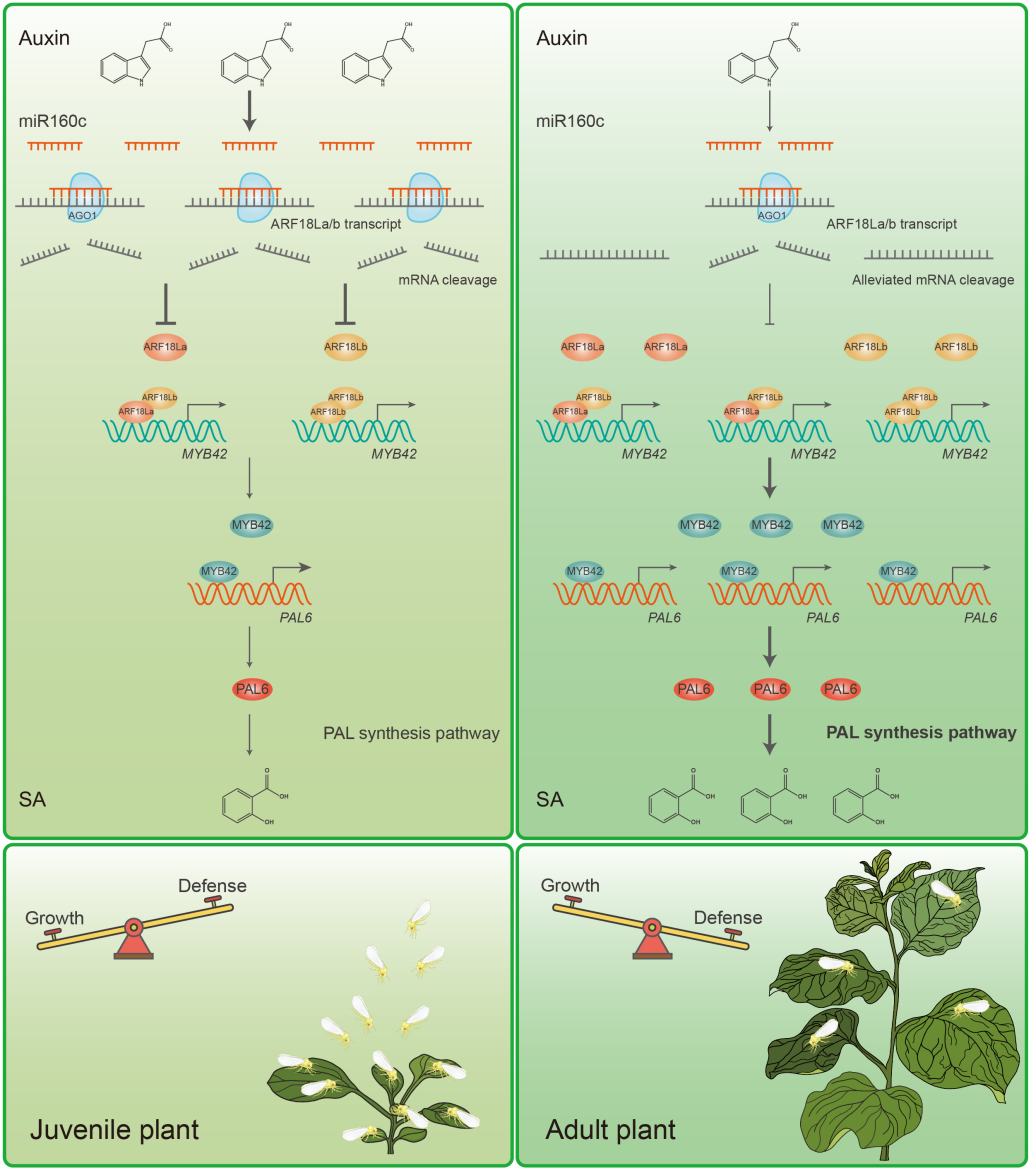
Auxin-SA seesaw mediates the age-related balance between plant growth and herbivore defense. At juvenile stages, plants exhibit high auxin levels, promoting the expression of *Nb*miR160c. This miRNA targets *NbARF18La/b* transcripts, leading to their degradation via the plant ARGONAUTE1 (AGO1) protein. As a result, NbARF18La/b is degraded at an early age, inhibiting downstream expression of *NbMYB42* and *NbPAL6*. This repression of the NbARF18La/b-NbMYB42-NbPAL6 module limits anti-whitefly SA accumulation in juvenile plants. As plants mature, the auxin levels decrease, lifting the repression of *NbARF18La/b*. These two interacting ARF TFs directly bind to the promoter of *NbMYB42*, activating its expression. NbMYB42 subsequently binds to the promoter of *NbPAL6*, enhancing its expression. NbPAL6 promotes SA biosynthesis, boosting SA-mediated defense against phloem-feeding insect whiteflies. The age-related rise in SA levels determines the ARR of plants. This model illustrates a seesaw-like mechanism for balancing growth and herbivore defense, with auxin dominance in early stages and SA-driven defense taking precedence as the plant ages.

Furthermore, we provide insights into why SA-mediated resistance is not fully established in juvenile plants. Specifically, high levels of auxin in juvenile plants activate *Nb*miR160c, which silences the *NbARF18La/b* genes, ultimately impairing SA accumulation. This mechanism also prevents excessive SA from disrupting early plant growth. Our findings deepen the understanding of the PVH, demonstrating that juvenile plants exhibit lower herbivore tolerance while prioritizing urgent growth needs (Fig. 9).

Our previous research has established the significant role of inducible SA in tobacco defense against whiteflies (*52*). In the current study, we found that the increased intrinsic SA levels associated with age also correlate with enhanced defense against whiteflies in adult plants (Fig. 1). To further investigate the role of SA-mediated ARR, we used a well-characterized NahG plant line. This NahG plant exhibited the same baseline SA levels as wild-type plants at the juvenile age but failed to accumulate additional SA with age or in response to whitefly infestation, unlike wild-type plants (Fig. 1, G and H). As a result, NahG plants displayed compromised defense capabilities at both juvenile and adult ages (Fig. 1I). Notably, exogenous SA application restored whitefly resistance in adult NahG plants (Fig. 1K), confirming the indispensable role of SA in ARR against whiteflies. At the same time, although JA is crucial for plant defense against whiteflies (*52*), our findings indicate that the intrinsic JA levels did not vary with age across several Solanaceae species (Fig. 1F), indicating that JA may not be a key phytohormonal signal in plant ARR against whiteflies. Recently, the role of SA in plant defense against phloem-feeding insects has gained attention (*53*–*55*), and our findings broaden the understanding of SA-mediated defenses from a unique perspective.

In *Arabidopsis*, the ICS pathway is primarily responsible for SA biosynthesis (*56*). However, a recent study revealed that ICS is not required for SA synthesis in rice (*57*). Previous research has also shown that ICS enzymes from different plant species have distinct biochemical properties, suggesting potential species-specific functions (*58*). A recent study reported that the ICS enzyme in *N. benthamiana*, the species examined in this study, exhibits no detectable enzymatic activity in the SA biosynthesis pathway (*59*). In contrast, accumulating evidence supports the involvement of *PAL* genes in SA biosynthesis across various plant species, including tobacco (*41*, *60*, *61*). These studies underscore the importance of PAL-mediated SA accumulation in defense against diverse biotic stresses, such as pathogens, viruses, and phloem-feeding insects. In this study, we found that the PAL pathway, rather than the ICS pathway, in SA biosynthesis changes at the transcriptional level with age in *N. benthamiana* (Fig. 2, B and C). We systematically analyzed *PAL* genes in *N. benthamiana* (fig. S2) and determined the age-related up-regulation of *NbPAL6* expression (Fig. 2C) and its role in age-related SA accumulation (Fig. 2E). Our conclusion does not imply that *NbPAL6* is the sole *NbPAL* gene responsible for SA synthesis in *N. benthamiana*, as other PALs may contribute redundantly to SA synthesis. We suspect that additional NbPALs may participate in SA accumulation under various conditions, including biotic or abiotic stresses, warranting further investigation. Furthermore, the relative contributions of the ICS and PAL pathways to SA synthesis in *N. benthamiana* and other plant species remain intriguing questions for future research. A more comprehensive understanding of these pathways will provide deeper insights into the biosynthetic origins and biological functions of phytohormones.

While extensive attention has been given to the upstream regulation of SA synthesis pathways, most studies have focused on the ICS pathway (*62*). The PAL pathway is known to contribute to the synthesis of numerous metabolic compounds in plants, such as flavonoids and lignin (*63*), but the regulation of *PAL* gene expression related to SA synthesis has been underexplored. For instance, in rice, the brown plant hopper induces OsMYB30, which activates *OsPAL* gene expression, leading to increased SA and lignin accumulation and enhanced resistance (*41*). In our study, we demonstrated that the TF NbMYB42 activates *NbPAL6* expression by binding to its promoter, thereby enhancing SA levels and whitefly defense in an age-related constitutive, but not inducible, manner (Fig. 3). In our previously published work, NbMYB42 was also shown to regulate lignin synthesis pathways in *N. benthamiana*, contributing to enhanced whitefly resistance (*42*). These observations combined highlight the necessity of MYB-PAL modules in plant SA and lignin synthesis, as well as their defense against phloem-feeding insects. In summary, our findings elucidate the mechanisms of age-related SA synthesis and regulation in plants, providing a valuable reference for future studies on the significance of age-enhanced SA in plant physiology and its induction in plant-pathogen and plant-insect interactions.

It is well established that SA and auxin signaling pathways often interact antagonistically (*15*). Elevated auxin levels correlate with reduced *PR* gene expression (*64*, *65*) and increased susceptibility to pathogens (*65*–*67*). In this study, we found that increased auxin levels can also affect plant defense against phloem-feeding insects by antagonizing SA levels (Fig. 5, A to F). Previous studies have indicated that auxin signaling may affect SA biosynthesis, although the underlying mechanisms remain largely unknown (*15*, *65*). Our study revealed that auxin inhibits the expression of the key synthesis gene *NbPAL* and its transcriptional regulators, NbMYB42 and NbARF18La/b, thereby reducing SA biosynthesis (Fig. 5, G to O). These findings provide valuable insights into the well-established antagonistic relationship between auxin and SA, highlighting a previously unrecognized mechanism involving biosynthetic and transcriptional regulation.

In addition, repression of auxin signaling by SA has been demonstrated in studies on plant growth. Plants that overaccumulate SA often exhibit morphological phenotypes reminiscent of auxin-deficient or auxin-insensitive mutants, indicating that SA may interfere with auxin responses. For example, SA treatment decreased biomass in *Arabidopsis*, an effect that was abolished in the *auxin resistant 3* (*axr3*) mutant background, which harbors an auxin-negative regulator (*68*). Previous research has shown that SA causes a global repression of auxin-related genes, thereby inhibiting auxin responses (*69*). Moreover, in *Arabidopsis*, SA suppresses auxin biosynthesis by inhibiting the H_2_O_2_-scavenging activity of catalases. This impairment increases H_2_O_2_ levels, which, in turn, leads to sulfenylation of Cys^308^ in the tryptophan synthetase β subunit 1 (TSB1), a key enzyme involved in the biosynthesis of the auxin precursor tryptophan, and reduced TSB1 activity (*70*). Similarly, we found that increased SA levels influenced auxin accumulation and related synthesis gene expression (Fig. 8, D to F), leading to reduced growth in *N. benthamiana* plants (Fig. 8, A to C). These findings deepen our understanding of the cross-talk between SA and auxin, particularly regarding upstream biosynthesis. However, the molecular mechanism by which SA suppresses *N. benthamiana* auxin biosynthesis genes remains to be elucidated. Furthermore, growing evidence supports the notion that the SA and auxin pathways act in a mutually antagonistic manner during plant immunity (*71*–*74*), with SA inhibiting auxin signaling as part of the immune response (*69*, *75*). In this study, we uncovered the critical physiological role of the SA-auxin feedback loop in balancing plant growth and insect defense (Fig. 9). These findings also underscore the importance of considering potential hormonal interactions when applying phytohormone-based strategies, such as SA treatments, to enhance crop pest tolerance. Factors such as plant age, environmental stress, and biotic stimuli should be carefully evaluated to avoid unintended growth trade-offs in agricultural practices.

Studies have shown that ARFs activate MYB TFs involved in various essential physiological processes (*76*–*79*). Our study complementarily reports that the two ARFs, NbARF18La and NbARF18Lb, positively regulate SA biosynthesis by promoting SA-related MYB TFs, thereby enhancing plant defense against herbivores (Fig. 4). Notably, the two ARFs we identified can interact with one another (fig. S5). Our findings indicate that auxin inhibits SA accumulation, positioning NbARFs as central hubs in this interaction. While ARFs are known participants in the antagonism between auxin and other phytohormones, such as abscisic acid and JA (*46*, *80*), little is reported regarding their relationship with SA. Previous studies have shown that the auxin-induced ARF7 represses the expression of SA-downstream *PR* genes that regulate bacterial infections in *Arabidopsis* plants (*81*). In contrast, we found that in *N. benthamiana*, auxin represses the transcriptional activators *NbARF18La/b* (Fig. 6), thereby negatively affecting upstream SA signaling.

In addition, this work reveals the mechanism by which auxin represses *NbARFs* through miR160c-mediated posttranscriptional regulation (Fig. 6). Prior studies have demonstrated that several miRNAs target *ARF* transcripts to regulate growth and development. For example, in *Arabidopsis*, miR160 targets *ARF10/16/17* (*82*), while miR167 antagonizes ARF6/8 (*50*). Our findings further illuminate the relatively unexplored role of miR160c in regulating *ARFs*, suggesting a mechanism by which it fine-tunes plant defense against phloem-feeding insects (Figs. 6 and 7).

Age-related changes in sRNA levels have been shown to play an important role in plant growth and stress responses. In *Arabidopsis*, the decline in the levels of miR156/7 with age drives plant maturation (*83*, *84*), while an increase in miR172 with plant age promotes the transition toward adulthood and flowering (*85*). Notably, age-related miRNA gradients are also implicated in plant immunity against pathogens. For example, miR172b levels are very low during the early stage of seedling development but increase over time, enhancing pathogen immunity by reducing the expression of *TARGET OF EAT1/2* (*TOE1/2*), repressors of the immune receptor *FLAGELLIN-SENSING2* (*FLS2*) (*86*). In this study, we demonstrate the role of age-related sRNA changes in regulating insect resistance. Specifically, the decrease in miRNA160c levels with age alleviates inhibition of its target defense-related TFs, thereby promoting insect resistance (Fig. 7).

The regulation of miRNA levels in plants is complex and influenced by various factors including phytohormones, which can either increase or decrease miRNA levels (*19*, *87*). We found that auxin increased miR160c levels by promoting its transcription (Fig. 7, J and K). The connections between miRNAs and phytohormones are an emerging area of research, with multiple recent studies highlighting the role of miRNAs in regulating plant phytohormonal pathways (*87*–*89*). As a supplementary finding, we revealed that auxin-induced miRNA can target key regulators involved in the antagonism of SA synthesis (Fig. 9). These findings broaden our understanding of the molecular and physiological mechanisms underlying phytohormone interactions. Our work demonstrates the role of miRNA in mediating the cross-talk between developmental auxin and defensive SA, balancing plant growth and defense (Fig. 9).

Developing new crop varieties with improved product traits and enhanced stress resilience is a primary goal of global crop breeding; however, this effort is often limited by the G-D trade-off (*89*). Consequently, the G-D trade-off has garnered increasing attention. However, the G-D balance in plants is not immutable; it can be influenced by various factors, such as developmental stage and environmental conditions. In this study, we specifically focused on the mechanisms underlying the trade-off between growth and insect resistance across different plant ages (Fig. 9). We conclude that the auxin-miR160c module represses the ARFs-MYB-PAL module, mediating insect defense while ensuring early growth during the juvenile stage. As plants mature, this repression is gradually alleviated, allowing for the establishment of SA-mediated resistance alongside continued plant growth. These findings are likely to inspire further strategies for regulating crop growth and stress resistance by fine-tuning phytohormone interactions, key TFs, metabolic pathways, and miRNA levels. Genetic optimization in breeding programs must fully consider the age dynamics of plant-insect interactions to overcome the G-D trade-offs and develop ideal crop varieties.

## MATERIALS AND METHODS

### Insect and plant materials

The *B. tabaci* MEAM1 whitefly (*mtCOI*, GenBank accession: GQ332577) was used in this study. Whiteflies were reared on cotton plants (*Gossypium hirsutum* cv. Zhemian 1793). Whitefly adults within 7 days postemergence were used for experiments. Green peach aphids (*M. persicae*) were reared on tobacco (*N. tabacum* cv. NC89), with adult winged aphids also taken at 7 days postemergence for experiments. The western flower thrips (*F. occidentalis*) were similarly reared on cotton plants (*G. hirsutum* cv. Zhemian 1793). Adult thrips, within 7 days postemergence, were taken in the experiments.

*N. benthamiana* seeds (accession LAB and line H2B-RFP), tobacco (*N. tabacum* cv. NC89), and tomato (*S. lycopersicum* cv. Hezuo903) were sown in a soil mixture composed of peat moss, perlite, and vermiculite in a ratio of 5:3:1 (v/v/v) and placed in climate chambers. After approximately 14 days, when the *N. benthamiana*, tobacco, and tomato seedlings had germinated in a seed starter tray, the resulting plants were transplanted into plastic pots for individual cultivation. *N. benthamiana* plants at 7 days posttransplanting (21 days old, three to four true leaves) were classified as juvenile plants (low resistance), while *N. benthamiana* plants at 21 days posttransplanting (35 days old, eight to nine true leaves) were referred to as adult plants (high resistance) (Fig. 2D). For tobacco, 7 days posttransplanting (21 days old, three to four true leaves) were classified as juvenile plants and 21 days pos-transplanting (35 days old, six to seven true leaves) were classified as adult plants. For tomatoes, 7 days posttransplanting (21 days old, three to four true leaves) were classified as juvenile plants and 21 days posttransplanting (35 days old, eight to nine true leaves) were classified as adult plants.

Insect-rearing plants and experimental plants were grown in climate chambers at Zhejiang University (Hangzhou, China) at 26° ± 1°C, 65 ± 10% relative humidity, and a photoperiod of 14-hour light:10-hour dark. All the insect-plant interaction experiments were conducted under the same conditions.

### Insect bioassay

Bioassays were conducted using the following methods and standards. The test plants were placed in specially designed nylon cages (30 cm–by–30 cm–by–30 cm, 120 mesh), and the test insects (five female and five male adults of MEAM1 whitefly, three female adults of aphid, and five female and five male adults of thrips, respectively) were released into each of the cages. The plants and insects used in the experimental study were chosen at random. After two days, the performance of insects (whitefly, survival rate and fecundity; aphid, offspring number; thrips, survival rate) was observed and recorded.

For the age-related bioassays, 21- and 35-day-old *N. benthamiana* plants were simultaneously prepared on the basis of their growth timeline. Specifically, we first sowed the initial batch of *N. benthamiana*, and when these plants reached 14 days old, we sowed the second batch. Both batches were grown in a strictly controlled environment as described above. By the time the first batch reached 35 days, the second batch was 21 days old. These two batches of *N. benthamiana* were then used for subsequent bioassays as mentioned above. Bioassays related to gene and miRNA functions were performed through *Agrobacterium*-mediated transient gene expression or VIGS. Two days after gene overexpressing or 14 days after gene silencing (Fig. 2D), the whiteflies were released onto the plants, and whitefly survival and fecundity were determined after 2 days. For bioassays involving the external application of chemicals to the plants, whitefly assessments commenced 2 days after chemical treatment.

### Transient expression assays

The full-length cDNAs of the selected genes (*NbPAL6*, *NbMYB42*, and *NbARF18Ls*) were amplified via PCR using gene-specific primer pairs (table S2). The full-length CDS sequences were inserted into pCAMBIA1305 vectors using the NovoRec plus one-step PCR cloning kit (Novoprotein, Suzhou, China) to generate gene expression constructs (pCAMBIA1305-*NbPAL6*-GFP, pCAMBIA1305-*NbMYB42*-GFP, pCAMBIA1305-*NbARF18La*-GFP, and pCAMBIA1305-*NbARF18Lb*-GFP). For transient expression in *N. benthamiana*, *Agrobacterium tumefaciens* strain EHA105 containing the respective vector was cultured overnight in LB medium at 28°C. Bacterial cultures were harvested by centrifugation, resuspended in buffer [10 mM MES (pH 5.7), 10 mM MgCl_2_, and 200 μM acetosyringone] to an optical density at 600 nm (OD_600_) of 0.8, and infiltrated into leaves of 21- or 35-day-old *N. benthamiana* using a needleless syringe (Fig. 2D). The infiltrated plants were then maintained in the previously described climate chambers. Two days later, the treated plants were used for follow-up experiments including bioassays and sample collection (Fig. 2D).

### VIGS assays

A 300-bp fragment of the target gene from *N. benthamiana* was identified using the VIGS tool on the Sol Genomics Network (https://vigs.solgenomics.net/). The gene fragment was amplified from *N. benthamiana* cDNA via PCR and cloned into the pTRV2 vector. The resulting plasmid was introduced into *A. tumefaciens* strain EHA105 by electroporation. Following cultivation, bacterial cells were harvested, resuspended, and adjusted to an OD_600_ of 0.2. The cultures were incubated at room temperature for at least 3 hours. For leaf infiltration, cultures containing pTRV1 were mixed in equal volumes with those containing pTRV2:00 (empty vector, control), pTRV2-*NbPAL6*, pTRV2-*NbMYB42*, or pTRV2-*NbARF18Ls*. These suspensions were infiltrated into 21-day-old *N. benthamiana* leaves using a 1-ml needleless syringe (Fig. 2D). Following infiltration, the *N. benthamiana* plants were maintained in the same climate chamber conditions. Fourteen days later, the treated plants were used for follow-up experiments, including bioassays and sample collection (Fig. 2D).

### Subcellular localization

The pCAMBIA1305-*NbMYB42*-GFP, pCAMBIA1305-*NbARF18La*-GFP, pCAMBIA1305-*NbARF18Lb*-GFP, and pCAMBIA1305-GFP (empty vector) constructs were used for subcellular localization. These constructs were infiltrated into the leaves of *N. benthamiana* line H2B-RFP via *A. tumefaciens* strain EHA105. Two days post-infiltration, GFP fluorescence signals were visualized using confocal microscopy (Zeiss LSM710, Oberkochen, Germany).

### RNA extraction and RT-qPCR analysis

Total RNA was extracted using AG RNAex Pro reagent (Accurate Biology, Changsha, China). For mRNA, single-stranded cDNA was synthesized using the *Evo M-MLV* RT Kit with gDNA Clean for qPCR Ver.2 (Accurate Biology Changsha, China). For plant miRNA cDNA synthesis, the miRNA 1st strand cDNA synthesis kit (Accurate Biology, Changsha, China) was used. RT-qPCR was conducted on a Bio-Rad CFX96 real-time PCR system (Bio-Rad, California, USA) with specific primers (table S2). Data were analyzed with Bio-Rad CFX Manager Software, using the 2^−∆∆C^_T_ method. The *N. benthamiana* housekeeping gene *NbACTIN* served as the internal control for gene expression analysis, and the expression of the *NbU6* snRNA genes was used as the internal control for miRNA level analysis.

### Measurement of phytohormone content

For the quantification of phytohormones (SA, JA, and IAA), *N. benthamiana* leaves were promptly excised and ground into powder in liquid nitrogen. Leaf powder (0.15 g) was mixed with 1 ml of high-performance liquid chromatography (HPLC)–grade ethyl acetate (Sinopharm, Shanghai, China) containing 10 ng of D4-SA (Quality Control Chemicals, Spokane, WA, USA), 10 ng of D6-JA (Quality Control Chemicals, Spokane, WA, USA), and 5 ng of D5-IAA (OlhemIm, Shanghai, China). The mixture was fully vortexed and centrifuged. The supernatant was collected and dried using a vacuum concentrator (Eppendorf, Hamburg, Germany) at 30°C. The residue was resuspended in 110 μl of methanol:H_2_O (50:50, v/v), and the resulting supernatant was analyzed via HPLC–tandem mass spectrometry (TripleTOF 5600+; AB Sciex, Redwood City, CA, USA).

### Degradome sequencing

The degradome sequencing analysis, performed according to established protocols from a previous study (*90*), was outsourced to Lianchuan Biotechnology Co. Ltd. Poly(A) RNA was purified from total plant RNA using poly-T oligo magnetic beads (Invitrogen, Carlsbad, CA, USA) through two rounds of purification. First-strand cDNA synthesis was performed with a 3′-adapter random primer, followed by size selection using AMPureXP beads (Beckman Coulter, Indianapolis, IN, USA) and PCR amplification. The final cDNA library had an average insert size of 200 to 400 bp. Sequencing was carried out with 50-bp single-end reads on an Illumina HiSeq 2500 (Illumina, San Diego, CA, USA). CleaveLand 3.0 and TargetFinder were used to identify potential sliced targets of known miRNAs.

### Y1H and Y2H assays

For the Y1H assay, approximately 2 kb of the promoter regions of the target genes (*NbPAL6* and *NbMYB42*) were inserted into the pAbAi vector to construct the bait plasmids. These plasmids were integrated into the Y1HGold yeast strain via polyethylene glycol (PEG)–mediated transformation, following in the Yeast User Manual PT4087-1 (Clontech, Shiga, Japan). The CDSs of the corresponding TFs *NbMYB42* and *NbARF18La/b* were cloned into the pGADT7 vector. The relevant vectors were coexpressed in Y1H yeast cells and screened with varying concentrations of Aureobasidin A (AbA, TaKaRa, Beijing, China).

For the Y2H assays, the full-length CDSs of the target genes *NbARF18La* and *NbARF18Lb* were ligated into pGBKT7 and pGADT7 (Clontech, Shiga, Japan), respectively. The paired vectors were transformed into Y2HGold yeast strain cells via PEG/LiAc transformation and grown on SD −Leu/−Trp medium. After 3 to 4 days of incubation, the clones were transferred onto SD −Leu/−Trp/−His/−Ade medium and incubated at 29°C for 3 to 4 days. The primers used for vector construction related to the Y1H and Y2H assays are listed in table S2.

### Histochemical GUS activity assays

For the reporter constructs, the promoters of the target genes *NbPAL6* and *NbMYB42* were introduced into the pBI121 vector containing a *GUS* gene–coding region. Simultaneously, the full-length CDS of the corresponding target TFs (*NbMYB42* and *NbARF18La/b*) were inserted into the pCAMBIA1305 vector to generate effector constructs (pCAMBIA1305-*NbMYB42*-GFP, pCAMBIA1305-*NbARF18La*-GFP, pCAMBIA1305-*NbARF18Lb*-GFP, as described in the transient expression assays section). The pCAMBIA1305 vector without the target CDS was used as the negative control (empty effector). These vectors were transformed into *A. tumefaciens* strain EHA105, and reporter vectors were cotransformed with the corresponding effector or control vectors for transient expression. GUS activity was assessed using a GUS histochemical assay kit (Coolaber, Beijing, China) according to the manufacturer’s instructions. GUS expression was visualized through staining and documented using an iPhone 13 Pro Max (Apple, California, USA). The experiment was repeated three to four times.

### Dual-luciferase assays

For the reporter construct, the promoters of the target genes (*NbPAL6* and *NbMYB42*) were inserted into the reporter vector pGreenII 0800-LUC. Effector constructs, including pCAMBIA1305-*NbMYB42*-GFP, pCAMBIA1305-*NbARF18La*-GFP, pCAMBIA1305-*NbARF18Lb*-GFP, and the empty vector pCAMBIA1305, were the same as those used in the histochemical GUS activity assays. These vectors were transformed into *A. tumefaciens* EHA105 (pSoup). In this system, *LUC* expression was driven by the target gene promoters, while *REN* was driven by the CaMV *35S* promoter as an internal control. The reporter and corresponding effector constructs were transiently transformed into *N. benthamiana* leaves. After a 3-day incubation, LUC signals were measured using the Dual-Luciferase Reporter Assay Kit (Vazyme, Nanjing, China) and analyzed on a FlexStation 3 Multi-Mode Microplate Reader (Molecular Devices, San Jose, CA, USA). Relative luciferase activity was calculated on the basis of LUC:REN ratios.

### ChIP assay

Leaves transformed with pCAMBIA1305-*NbMYB42*-GFP, pCAMBIA1305-*NbARF18La*-GFP, or pCAMBIA1305-*NbARF18Lb*-GFP (as described in the transient expression assay), were collected for ChIP assays. The experiment followed established protocols using the same transient gene expression system (*91*). Briefly, transformed *N. benthamiana* leaves were harvested and fixed with 1% formaldehyde. The fixation was quenched by adding 1 × phosphate-buffered saline (PBS) buffer containing 0.125 M glycine under vacuum for 5 min. Chromatin DNA was sonicated to fragments of approximately 200 to 300 bp. The chromatin complexes were immunoprecipitated by Protein G Agarose (Beyotime, Shanghai, China) with an anti-GFP monoclonal antibody (AG281, Beyotime, Shanghai, China). The coimmunoprecipitated DNA was recovered at 65°C, purified, and analyzed by qPCR with SYBR qPCR Mix (Accurate Biology, Changsha, China) with gene-specific primers (table S2). The relative fold enrichment was calculated using leaves transformed with the pCAMBIA1305-GFP (empty vector) as a control.

### EMSA assay

To prepare the test TF proteins for EMSA, the CDS of *NbMYB42* was fused into the GST-tagged pGEX-6P-1 vector to generate NbMYB42-GST. The *NbARF18La* CDS was fused into the His-tagged pET28a-SUMO vector, generating NbARF18La-His-SUMO, and the *NbARF18Lb* CDS was fused into the MBP-tagged pMAL-c5x vector, generating NbARF18Lb-MBP. These fusion proteins were expressed in *E. coli* BL21 cells using 0.2 mM isopropyl-β-d-thiogalactopyranoside and purified. For protein purification, NbMYB42-GST was purified through GST-affinity chromatography (Beyotime, Shanghai, China), NbARF18Lb-MBP using MBPSep Dextrin Agarose Resin 6FF (Yeasen, Shanghai, China), and NbARF18La-His with Ni–nitrilotriacetic acid Beads 6FF (Smart-Lifesciences, Changzhou, China).

Putative binding sites in the target gene promoters were predicted by Jaspar (https://jaspar.elixir.no/). For the *NbMYB42* and *NbPAL6* promoter interaction, biotin-labeled probes were designed on the basis of the P3 sequence (GGTGGTTGTTGAGAGG) of the *NbPAL6* promoter (Fig. 3G and table S2), with a mutated version (AAAAAAAAAAAAAAAA) serving as the mutant probe. For the interaction between NbARF18La/b and the *NbMYB42* promoter, biotin-labeled probes targeting the AuxRE element P2 sequence (TGTCTC) were designed (Fig. 4, G and H, and table S2), with a mutated version (AAAAAA) as the control. Cold competitors (10-, 20-, and 50-fold excess) of unlabeled probes were included in the reactions. Probes were labeled using the EMSA Probe Biotin Labeling Kit (Beyotime, Shanghai, China). Approximately 300 ng of the fusion protein was incubated with 30 ng of labeled probes, and specific binding was assessed via the Chemiluminescent EMSA Kit (Beyotime, Shanghai, China) following the manufacturer’s instructions. Chemiluminescent imaging was performed with the ChemiDoc Touch system (Bio-Rad, California, USA). Labeled probes incubated with GST, His-SUMO, and MBP proteins served as negative controls.

### Pull-down assay

Pull-down assays were conducted to determine the interaction of NbARF18La and NbARF18Lb. The recombinant proteins NbARF18La-His and NbARF18Lb-MBP obtained in the abovementioned EMSA assay were further used in this test. For MBP pull-down, MBPSep Dextrin Agarose Resin (Yeasen, Shanghai, China) is prepared by washing them with PBS and then incubating them with MBP-bait protein NbARF18Lb or control MBP protein. After washing, the beads are incubated with His-tagged interacting protein NbARF18La at 4°C for 3 hours. Then, the MBP beads underwent four washes with washing buffer [20 mM phosphate buffer, 500 mM NaCl, and 100 mM imidazole (pH 7.4)] and were then collected using collecting buffer [20 mM phosphate buffer, 500 mM NaCl, 500 mM imidazole (pH 7.4)]. The eluted samples were separated by SDS–polyacrylamide gel electrophoresis and detected by an anti-MBP (66003-1-Ig, Proteintech, Wuhan, China) antibody and an anti-His antibody (66005-1-Ig, Proteintech, Wuhan, China).

### BiFC assay

The CDSs of *NbARF18La* and *NbARF18Lb* were inserted into p2YN and p2YC vectors, respectively. *A. tumefaciens* EHA105 cells carrying p2YN constructs were combined with cells containing p2YC constructs at a 1:1 ratio. This mixture was then infiltrated into the leaves of the *N. benthamiana* H2B-RFP line. After 3 days of incubation, fluorescence was visualized under a laser confocal microscope (Zeiss LSM710, Oberkochen, Germany).

### Exogenous application of phytohormones

The target compound was dissolved in methanol (for SA, IAA, and NAA) or dimethyl sulfoxide (for Yucasin and L-Kyn) to produce the stock solution. Lanolin was selected as a slow-release medium. The lanolin was heated to a liquid state, and the stock solution was added to the lanolin to create the final mixture: SA at 1 mM; IAA and NAA at 1, 25, and 50 μM; Yucasin and L-Kyn at 50 μM. All the compounds were purchased from Aladdin, Shanghai, China. The lanolin solution containing the target compounds was transferred to a 1-ml sterile syringe and stored at −20°C.

Exogenous SA was applied to 21- and 35-day-old *N. benthamiana* plants. IAA and NAA were applied to 35-day-old plants, while Yucasin and _L_-Kyn were applied to 21-day-old plants. For application, 0.25 ml of lanolin was squeezed from the syringe and gently smeared along the midvein at the base of the leaves using latex-gloved fingers. After 2 days (Fig. 2D), the treated plants were used in bioassays or for sample collection for further experiments.

### Transient expression and functional inhibition of miRNA in *N. benthamiana*

To overexpress target miRNA *Nb*mi160c in *N. benthamiana*, the precursor sequences of *Nb*miR160c were synthesized by GenScript (Nanjing, China) and inserted into the pCAMBIA1300 vector through specific primers (table S2 and fig. S8A). The constructs were infiltrated into 35-day-old *N. benthamiana* plants, and after 2 days (Fig. 2D), the treated plants were used in bioassays or for sample collection for further experiments.

To inhibit the *Nb*miR160c function, a target mimicry (mimic160c) expression system was introduced. The precursor sequences of mimic160c were synthesized by GenScript (Nanjing, China) and inserted into the pCAMBIA1300 vector using specific primers (table S2 and fig. S8F). The constructs were infiltrated into 21-day-old *N. benthamiana* plants, and after 2 days (Fig. 2D), the treated plants were used for bioassays or sample collection for further experiments.

### MiRNA targeting validation

GFP, GUS, and LUC reporter systems were used to investigate the cleavage of *NbARF18La* and *NbARF18Lb* targets by *Nb*miR160c. For the GFP reporter, the *Nb*miR160c target site of *NbARF18La* and *NbARF18Lb* mRNA, as well as its mutant form (fig. S8C) were cloned into the pCAMBIA1305-GFP vector to construct GFP sensors through specific primers (table S2 and fig. S8D). The constructs of the target site and mutant target site were coinfiltrated with pCAMBIA1300-*Nb*miR160c through *A. tumefaciens* strain EHA105. After 2 days of infiltration, GFP fluorescence was observed using confocal microscopy, as described above. For the GUS and LUC reporters, the *Nb*miR160c target site of *NbARF18La* and *NbARF18Lb* mRNA, along with its mutant form, were cloned into the pCAMBIA121-GUS and pGreenII 0800-LUC vectors, respectively, to construct GUS and LUC sensor through specific primers (table S2 and fig. S8D). These constructs were coinfiltrated with pCAMBIA1300-*Nb*miR160c. After 2 days of infiltration, GUS and LUC activity was examined using the previously described method.

### MiRNA dual-luciferase assay

Coexpression of the LUC reporter and *Nb*miR160c was carried out to evaluate the influence of *Nb*miR160c on downstream genes. The effectors pCAMBIA1300-*pre*-*Nb*miR160c, pCAMBIA1300-*pre*-mimic160c, and pCAMBIA1300 (empty vector control) were coexpressed with LUC reporters linked to the *NbMYB42* or *NbPAL6* promoters. After 2 days of infiltration, LUC activity was examined using the method mentioned method. The combination of effectors and reporters is shown in fig. S9 (A to D).

The system was also used to evaluate the possible influence of *Nb*miR160c on *NbPAL6* transcription when *NbARFs* were overexpressed. The *LUC* gene linked to the *NbPAL6* promoter or *NbMYB42* promoter was used as the reporter respectively. pCAMBIA1305-*NbARFs* plus pCAMBIA1300-*pre*-*Nb*miR160c, and pCAMBIA1305-*NbARFs* plus pCAMBIA1300 (empty vector) were coinfiltrated with the reporter into the leaves of *N. benthamiana*. LUC activity for the different combinations was detected and compared. The combinations of effectors and reporters are shown in Fig. 7G and fig. S9E.

### Statistical analysis

All data were analyzed using IBM SPSS Statistics 22.0 and visualized using GraphPad Prism 9. Statistical significance was determined using Student’s *t* test (two-tailed) or one-way analysis of variance (ANOVA), followed by Fisher’s least significant difference test.

## References

[1] D. Cipollini, C. B. Purrington, J. Bergelson, Costs of induced responses in plants. Basic Appl. Ecol. 4, 79–89 (2003).

[2] D. Walters, M. Heil, Costs and trade-offs associated with induced resistance. Physiol. Mol. Plant Pathol. 71, 3–17 (2007).

[3] G. A. Howe, G. Jander, Plant immunity to insect herbivores. Annu. Rev. Plant Biol. 59, 41–66 (2008). 18031220 10.1146/annurev.arplant.59.032607.092825

[4] M. Erb, P. Reymond, Molecular interactions between plants and insect herbivores. Annu. Rev. Plant Biol. 70, 527–557 (2019). 30786233 10.1146/annurev-arplant-050718-095910

[5] A. R. Zangerl, C. E. Rutledge, The probability of attack and patterns of constitutive and induced defense: A test of optimal defense theory. Am. Nat. 147, 599–608 (1996).

[6] J. A. Gatehouse, Plant resistance towards insect herbivores: A dynamic interaction. New Phytol. 156, 145–169 (2002). 33873279 10.1046/j.1469-8137.2002.00519.x

[7] P. W. Price, The plant vigor hypothesis and herbivore attack. Oikos 62, 244–251 (1991).

[8] S. W. Makhabu, C. Skarpe, H. Hytteborn, Z. D. Mpofu, The plant vigour hypothesis revisited – How is browsing by ungulates and elephant related to woody species growth rate? Plant Ecol. 184, 163–172 (2006).

[9] M.-P. Develey-Rivière, E. Galiana, Resistance to pathogens and host developmental stage: A multifaceted relationship within the plant kingdom. New Phytol. 175, 405–416 (2007). 17635216 10.1111/j.1469-8137.2007.02130.x

[10] J. H. Kim, H. R. Woo, J. Kim, P. O. Lim, I. C. Lee, S. H. Choi, D. Hwang, H. G. Nam, Trifurcate feed-forward regulation of age-dependent cell death involving *miR164* in *Arabidopsis*. Science 323, 1053–1057 (2009). 19229035 10.1126/science.1166386

[11] Y.-B. Mao, Y.-Q. Liu, D.-Y. Chen, F.-Y. Chen, X. Fang, G.-J. Hong, L.-J. Wang, J.-W. Wang, X.-Y. Chen, Jasmonate response decay and defense metabolite accumulation contributes to age-regulated dynamics of plant insect resistance. Nat. Commun. 8, 13925 (2017). 28067238 10.1038/ncomms13925 PMC5233801

[12] L. Hu, L. Yang, Time to fight: Molecular mechanisms of age-related resistance. Phytopathology 109, 1500–1508 (2019). 31192748 10.1094/PHYTO-11-18-0443-RVW

[13] T. Rankenberg, B. Geldhof, H. van Veen, K. Holsteens, B. Van de Poel, R. Sasidharan, Age-dependent abiotic stress resilience in plants. Trends Plant Sci. 26, 692–705 (2021). 33509699 10.1016/j.tplants.2020.12.016

[14] Z. He, S. Webster, S. Y. He, Growth-defense trade-offs in plants. Curr. Biol. 32, R634–R639 (2022). 35728544 10.1016/j.cub.2022.04.070

[15] A. Robert-Seilaniantz, M. Grant, J. D. G. Jones, Hormone crosstalk in plant disease and defense: More than just jasmonate-salicylate antagonism. Annu. Rev. Phytopathol. 49, 317–343 (2011). 21663438 10.1146/annurev-phyto-073009-114447

[16] M. Vanstraelen, E. Benková, Hormonal interactions in the regulation of plant development. Annu. Rev. Cell Dev. Biol. 28, 463–487 (2012). 22856461 10.1146/annurev-cellbio-101011-155741

[17] J. Curaba, M. B. Singh, P. L. Bhalla, miRNAs in the crosstalk between phytohormone signalling pathways. J. Exp. Bot. 65, 1425–1438 (2014). 24523503 10.1093/jxb/eru002

[18] M. L. Berens, H. M. Berry, A. Mine, C. T. Argueso, K. Tsuda, Evolution of hormone signaling networks in plant defense. Annu. Rev. Phytopathol. 55, 401–425 (2017). 28645231 10.1146/annurev-phyto-080516-035544

[19] X. Song, Y. Li, X. Cao, Y. Qi, MicroRNAs and their regulatory roles in plant-environment interactions. Annu. Rev. Plant Biol. 70, 489–525 (2019). 30848930 10.1146/annurev-arplant-050718-100334

[20] M. Altmann, S. Altmann, P. A. Rodriguez, B. Weller, L. Elorduy Vergara, J. Palme, N. Marín-de la Rosa, M. Sauer, M. Wenig, J. A. Villaécija-Aguilar, J. Sales, C.-W. Lin, R. Pandiarajan, V. Young, A. Strobel, L. Gross, S. Carbonnel, K. G. Kugler, A. Garcia-Molina, G. W. Bassel, C. Falter, K. F. X. Mayer, C. Gutjahr, A. C. Vlot, E. Grill, P. Falter-Braun, Extensive signal integration by the phytohormone protein network. Nature 583, 271–276 (2020). 32612234 10.1038/s41586-020-2460-0

[21] S.-S. Liu, P. J. De Barro, J. Xu, J.-B. Luan, L.-S. Zang, Y.-M. Ruan, F.-H. Wan, Asymmetric mating interactions drive widespread invasion and displacement in a whitefly. Science 318, 1769–1772 (2007). 17991828 10.1126/science.1149887

[22] P. J. De Barro, S.-S. Liu, L. M. Boykin, A. B. Dinsdale, *Bemisia tabaci*: A statement of species status. Annu. Rev. Entomol. 56, 1–19 (2011). 10.1146/annurev-ento-112408-085504 20690829

[23] S.-S. Liu, J. Colvin, P. J. De Barro, Species concepts as applied to the whitefly *Bemisia tabaci* systematics: How many species are there? J. Integr. Agric. 11, 176–186 (2012).

[24] S. Kanakala, M. Ghanim, Global genetic diversity and geographical distribution of *Bemisia tabaci* and its bacterial endosymbionts. PLOS ONE 14, e0213946 (2019). 30889213 10.1371/journal.pone.0213946 PMC6424426

[25] M. Inbar, D. Gerling, Plant-mediated interactions between whiteflies, herbivores, and natural enemies. Annu. Rev. Entomol. 53, 431–448 (2008). 17877454 10.1146/annurev.ento.53.032107.122456

[26] X.-W. Wang, P. Li, S.-S. Liu, Whitefly interactions with plants. Curr. Opin. Insect Sci. 19, 70–75 (2017). 28521945 10.1016/j.cois.2017.02.001

[27] S. Morin, P. W. Atkinson, L. L. Walling, Whitefly-plant interactions: An integrated molecular perspective. Annu. Rev. Entomol. 69, 503–525 (2024). 37816261 10.1146/annurev-ento-120120-093940

[28] X.-W. Wang, S. Blanc, Insect transmission of plant single-stranded DNA viruses. Annu. Rev. Entomol. 66, 389–405 (2021). 32931313 10.1146/annurev-ento-060920-094531

[29] M. M. Goodin, D. Zaitlin, R. A. Naidu, S. A. Lommel, *Nicotiana benthamiana*: Its history and future as a model for plant-pathogen interactions. Mol. Plant Microbe Interact. 21, 1015–1026 (2008). 18616398 10.1094/MPMI-21-8-1015

[30] J. Bally, H. Jung, C. Mortimer, F. Naim, J. G. Philips, R. Hellens, A. Bombarely, M. M. Goodin, P. M. Waterhouse, The rise and rise of *Nicotiana benthamiana*: A plant for all reasons. Annu. Rev. Phytopathol. 56, 405–426 (2018). 30149789 10.1146/annurev-phyto-080417-050141

[31] W.-H. Han, J.-X. Wang, F.-B. Zhang, S.-X. Ji, Y.-W. Zhong, Y.-Q. Liu, S.-S. Liu, X.-W. Wang, A new feature of the laboratory model plant *Nicotiana benthamiana*: Dead-end trap for sustainable field pest control. Plants People Planet 6, 743–759 (2024).

[32] J.-X. Wang, W.-H. Han, R. Xie, F.-B. Zhang, Z.-W. Ge, S.-X. Ji, S.-S. Liu, X.-W. Wang, Metabolic and molecular insights into *Nicotiana benthamiana* trichome exudates: An ammunition depot for plant resistance against insect pests. Plant Cell Environ. 48, 387–405 (2025). 39262218 10.1111/pce.15135

[33] X.-Y. Zhang, W.-H. Han, F.-B. Zhang, J.-X. Wang, S.-S. Liu, X.-W. Wang, Attraction of *Nicotiana benthamiana* to *Bemisia tabaci* is related to a chemical signal in plant volatile, undecane. Pest Manag. Sci. (2024); https://doi.org/10.1002/ps.8411. 10.1002/ps.8411 39258464

[34] H. Chen, M. Li, G. Qi, M. Zhao, L. Liu, J. Zhang, G. Chen, D. Wang, F. Liu, Z. Q. Fu, Two interacting transcriptional coactivators cooperatively control plant immune responses. Sci. Adv. 7, eabl7173 (2021). 34739308 10.1126/sciadv.abl7173 PMC8570602

[35] H. Wang, S. Song, S. Gao, Q. Yu, H. Zhang, X. Cui, J. Fan, X. Xin, Y. Liu, B. Staskawicz, T. Qi, The NLR immune receptor ADR1 and lipase-like proteins EDS1 and PAD4 mediate stomatal immunity in *Nicotiana benthamiana* and Arabidopsis. Plant Cell 36, 427–446 (2024). 37851863 10.1093/plcell/koad270 PMC10827572

[36] P. Zhang, J. Li, X. Gou, L. Zhu, Y. Yang, Y. Li, Y. Zhang, L. Ding, A. Ansabayeva, Y. Meng, W. Shan, The *Phytophthora infestans* effector Pi05910 suppresses and destabilizes host glycolate oxidase StGOX4 to promote plant susceptibility. Mol. Plant Pathol. 25, e70021 (2024). 39487604 10.1111/mpp.70021 PMC11530570

[37] T. Gaffney, L. Friedrich, B. Vernooij, D. Negrotto, G. Nye, S. Uknes, E. Ward, H. Kessmann, J. Ryals, Requirement of salicylic acid for the induction of systemic acquired resistance. Science 261, 754–756 (1993). 17757215 10.1126/science.261.5122.754

[38] Y. Peng, J. Yang, X. Li, Y. Zhang, Salicylic acid: Biosynthesis and signaling. Annu. Rev. Plant Biol. 72, 761–791 (2021). 33756096 10.1146/annurev-arplant-081320-092855

[39] C. J. Arntzen, Using tobacco to treat cancer. Science 321, 1052–1053 (2008). 18719274 10.1126/science.1163420

[40] B. Craven-Bartle, M. B. Pascual, F. M. Cánovas, C. Avila, A Myb transcription factor regulates genes of the phenylalanine pathway in maritime pine. Plant J. 74, 755–766 (2013). 23451763 10.1111/tpj.12158

[41] J. He, Y. Liu, D. Yuan, M. Duan, Y. Liu, Z. Shen, C. Yang, Z. Qiu, D. Liu, P. Wen, J. Huang, D. Fan, S. Xiao, Y. Xin, X. Chen, L. Jiang, H. Wang, L. Yuan, J. Wan, An R2R3 MYB transcription factor confers brown planthopper resistance by regulating the phenylalanine ammonia-lyase pathway in rice. Proc. Natl. Acad. Sci. U.S.A. 117, 271–277 (2020). 31848246 10.1073/pnas.1902771116 PMC6955232

[42] F.-B. Zhang, S.-X. Ji, J.-G. Yang, X.-W. Wang, W.-H. Han, Genome-wide analysis of MYB family in *Nicotiana benthamiana* and the functional role of the key members in resistance to *Bemisia tabaci*. Int. J. Biol. Macromol. 235, 123759 (2023). 36812971 10.1016/j.ijbiomac.2023.123759

[43] D. R. Boer, A. Freire-Rios, W. A. M. van den Berg, T. Saaki, I. W. Manfield, S. Kepinski, I. López-Vidrieo, J. M. Franco-Zorrilla, S. C. de Vries, R. Solano, D. Weijers, M. Coll, Structural basis for DNA binding specificity by the auxin-dependent ARF transcription factors. Cell 156, 577–589 (2014). 24485461 10.1016/j.cell.2013.12.027

[44] T. J. Guilfoyle, The PB1 domain in auxin response factor and Aux/IAA proteins: A versatile protein interaction module in the auxin response. Plant Cell 27, 33–43 (2015). 25604444 10.1105/tpc.114.132753 PMC4330575

[45] D. A. Korasick, S. Chatterjee, M. Tonelli, H. Dashti, S. G. Lee, C. S. Westfall, D. B. Fulton, A. H. Andreotti, G. K. Amarasinghe, L. C. Strader, J. M. Jez, Defining a two-pronged structural model for PB1 (Phox/Bem1p) domain interaction in plant auxin responses. J. Biol. Chem. 290, 12868–12878 (2015). 25839233 10.1074/jbc.M115.648253 PMC4432302

[46] C. Cancé, R. Martin-Arevalillo, K. Boubekeur, R. Dumas, Auxin response factors are keys to the many auxin doors. New Phytol. 235, 402–419 (2022). 35434800 10.1111/nph.18159

[47] S.-B. Li, Z.-Z. Xie, C.-G. Hu, J.-Z. Zhang, A review of auxin response factors (ARFs) in plants. Front. Plant Sci. 7, 47 (2016). 26870066 10.3389/fpls.2016.00047 PMC4737911

[48] K. Jiang, T. Asami, Chemical regulators of plant hormones and their applications in basic research and agriculture. Biosci. Biotechnol. Biochem. 82, 1265–1300 (2018). 29678122 10.1080/09168451.2018.1462693

[49] E. Marin, V. Jouannet, A. Herz, A. S. Lokerse, D. Weijers, H. Vaucheret, L. Nussaume, M. D. Crespi, A. Maizel, miR390, *Arabidopsis TAS3* tasiRNAs, and their *AUXIN RESPONSE FACTOR* targets define an autoregulatory network quantitatively regulating lateral root growth. Plant Cell 22, 1104–1117 (2010). 20363771 10.1105/tpc.109.072553 PMC2879756

[50] N. Liu, S. Wu, J. Van Houten, Y. Wang, B. Ding, Z. Fei, T. H. Clarke, J. W. Reed, E. van der Knaap, Down-regulation of *AUXIN RESPONSE FACTORS 6* and *8* by microRNA 167 leads to floral development defects and female sterility in tomato. J. Exp. Bot. 65, 2507–2520 (2014). 24723401 10.1093/jxb/eru141 PMC4036516

[51] J.-J. Wang, H.-S. Guo, Cleavage of INDOLE-3-ACETIC ACID INDUCIBLE28 mRNA by microRNA847 upregulates auxin signaling to modulate cell proliferation and lateral organ growth in Arabidopsis. Plant Cell 27, 574–590 (2015). 25794935 10.1105/tpc.15.00101 PMC4558675

[52] Y.-X. Liu, W.-H. Han, J.-X. Wang, F.-B. Zhang, S.-X. Ji, Y.-W. Zhong, S.-S. Liu, X.-W. Wang, Differential induction of JA/SA determines plant defense against successive leaf-chewing and phloem-feeding insects. J. Pest Sci., 1–16 (2024).

[53] T. Züst, A. A. Agrawal, Mechanisms and evolution of plant resistance to aphids. Nat. Plants 2, 15206 (2016). 27250753 10.1038/nplants.2015.206

[54] R. Blundell, J. E. Schmidt, A. Igwe, A. L. Cheung, R. L. Vannette, A. C. M. Gaudin, C. L. Casteel, Organic management promotes natural pest control through altered plant resistance to insects. Nat. Plants 6, 483–491 (2020). 32415295 10.1038/s41477-020-0656-9

[55] D. Mertens, M. Fernández de Bobadilla, Q. Rusman, J. Bloem, J. C. Douma, E. H. Poelman, Plant defence to sequential attack is adapted to prevalent herbivores. Nat. Plants 7, 1347–1353 (2021). 34650263 10.1038/s41477-021-00999-7

[56] D. Rekhter, D. Lüdke, Y. Ding, K. Feussner, K. Zienkiewicz, V. Lipka, M. Wiermer, Y. Zhang, I. Feussner, Isochorismate-derived biosynthesis of the plant stress hormone salicylic acid. Science 365, 498–502 (2019). 31371615 10.1126/science.aaw1720

[57] Z. Wang, G. Yang, D. Zhang, G. Li, J.-L. Qiu, J. Wu, Isochorismate synthase is required for phylloquinone, but not salicylic acid biosynthesis in rice. aBIOTECH 5, 488–496 (2024). 39650133 10.1007/s42994-024-00166-4 PMC11624176

[58] S. Yokoo, S. Inoue, N. Suzuki, N. Amakawa, H. Matsui, H. Nakagami, A. Takahashi, R. Arai, S. Katou, Comparative analysis of plant isochorismate synthases reveals structural mechanisms underlying their distinct biochemical properties. Biosci. Rep. 38, BSR20171457 (2018). 29436485 10.1042/BSR20171457 PMC5843753

[59] Z. Su, C. Niu, S. Zhou, G. Xu, P. Zhu, Q. Fu, Y. Zhang, Z. Ming, Structural basis of chorismate isomerization by *Arabidopsis* ISOCHORISMATE SYNTHASE1. Plant Physiol. 196, 773–787 (2024). 38701037 10.1093/plphys/kiae260

[60] D. Ogawa, N. Nakajima, S. Seo, I. Mitsuhara, H. Kamada, Y. Ohashi, The phenylalanine pathway is the main route of salicylic acid biosynthesis in *Tobacco mosaic virus*-infected tobacco leaves. Plant Biotechnol. 23, 395–398 (2006).

[61] M. B. Shine, J.-W. Yang, M. El-Habbak, P. Nagyabhyru, D.-Q. Fu, D. Navarre, S. Ghabrial, P. Kachroo, A. Kachroo, Cooperative functioning between phenylalanine ammonia lyase and isochorismate synthase activities contributes to salicylic acid biosynthesis in soybean. New Phytol. 212, 627–636 (2016). 27411159 10.1111/nph.14078

[62] P. Ding, Y. Ding, Stories of salicylic acid: A plant defense hormone. Trends Plant Sci. 25, 549–565 (2020). 32407695 10.1016/j.tplants.2020.01.004

[63] N.-Q. Dong, H.-X. Lin, Contribution of phenylpropanoid metabolism to plant development and plant-environment interactions. J. Integr. Plant Biol. 63, 180–209 (2021). 33325112 10.1111/jipb.13054

[64] J.-E. Park, J.-Y. Park, Y.-S. Kim, P. E. Staswick, J. Jeon, J. Yun, S.-Y. Kim, J. Kim, Y.-H. Lee, C.-M. Park, GH3-mediated auxin homeostasis links growth regulation with stress adaptation response in Arabidopsis. J. Biol. Chem. 282, 10036–10046 (2007). 17276977 10.1074/jbc.M610524200

[65] A. Robert-Seilaniantz, D. MacLean, Y. Jikumaru, L. Hill, S. Yamaguchi, Y. Kamiya, J. D. G. Jones, The microRNA miR393 re-directs secondary metabolite biosynthesis away from camalexin and towards glucosinolates. Plant J. 67, 218–231 (2011). 21457368 10.1111/j.1365-313X.2011.04591.x

[66] L. Navarro, R. Bari, P. Achard, P. Lisón, A. Nemri, N. P. Harberd, J. D. G. Jones, DELLAs control plant immune responses by modulating the balance of jasmonic acid and salicylic acid signaling. Curr. Biol. 18, 650–655 (2008). 18450451 10.1016/j.cub.2008.03.060

[67] S. A. McClerklin, S. G. Lee, C. P. Harper, R. Nwumeh, J. M. Jez, B. N. Kunkel, Indole-3-acetaldehyde dehydrogenase-dependent auxin synthesis contributes to virulence of Pseudomonas syringae strain DC3000. PLOS Pathog. 14, e1006811 (2018). 29293681 10.1371/journal.ppat.1006811 PMC5766252

[68] J. V. Canet, A. Dobón, F. Ibáñez, L. Perales, P. Tornero, Resistance and biomass in Arabidopsis: A new model for salicylic acid perception. Plant Biotechnol. J. 8, 126–141 (2010). 20040060 10.1111/j.1467-7652.2009.00468.x

[69] D. Wang, K. Pajerowska-Mukhtar, A. H. Culler, X. Dong, Salicylic acid inhibits pathogen growth in plants through repression of the auxin signaling pathway. Curr. Biol. 17, 1784–1790 (2007). 17919906 10.1016/j.cub.2007.09.025

[70] H.-M. Yuan, W.-C. Liu, Y.-T. Lu, CATALASE2 coordinates SA-mediated repression of both auxin accumulation and JA biosynthesis in plant defenses. Cell Host Microbe 21, 143–155 (2017). 28182949 10.1016/j.chom.2017.01.007

[71] K. Kazan, J. M. Manners, Linking development to defense: Auxin in plant-pathogen interactions. Trends Plant Sci. 14, 373–382 (2009). 19559643 10.1016/j.tplants.2009.04.005

[72] C. An, Z. Mou, Salicylic acid and its function in plant immunity. J. Integr. Plant Biol. 53, 412–428 (2011). 21535470 10.1111/j.1744-7909.2011.01043.x

[73] A. M. Mutka, S. Fawley, T. Tsao, B. N. Kunkel, Auxin promotes susceptibility to *Pseudomonas syringae* via a mechanism independent of suppression of salicylic acid-mediated defenses. Plant J. 74, 746–754 (2013). 23521356 10.1111/tpj.12157

[74] H. Tian, L. Xu, X. Li, Y. Zhang, Salicylic acid: The roles in plant immunity and crosstalk with other hormones. J. Integr. Plant Biol. 67, 773–785 (2025). 39714102 10.1111/jipb.13820 PMC11951402

[75] S. Tan, M. Abas, I. Verstraeten, M. Glanc, G. Molnár, J. Hajný, P. Lasák, I. Petřík, E. Russinova, J. Petrášek, O. Novák, J. Pospíšil, J. Friml, Salicylic acid targets protein phosphatase 2A to attenuate growth in plants. Curr. Biol. 30, 381–395.e8 (2020). 31956021 10.1016/j.cub.2019.11.058 PMC6997888

[76] R. Ghelli, P. Brunetti, N. Napoli, A. De Paolis, V. Cecchetti, T. Tsuge, G. Serino, M. Matsui, G. Mele, G. Rinaldi, G. A. Palumbo, F. Barozzi, P. Costantino, M. Cardarelli, A newly identified flower-specific splice variant of *AUXIN RESPONSE FACTOR8* regulates stamen elongation and endothecium lignification in Arabidopsis. Plant Cell 30, 620–637 (2018). 29514943 10.1105/tpc.17.00840 PMC5894849

[77] X.-F. Xu, B. Wang, Y.-F. Feng, J.-S. Xue, X.-X. Qian, S.-Q. Liu, J. Zhou, Y.-H. Yu, N.-Y. Yang, P. Xu, Z.-N. Yang, AUXIN RESPONSE FACTOR17 directly regulates *MYB108* for anther dehiscence. Plant Physiol. 181, 645–655 (2019). 31345954 10.1104/pp.19.00576 PMC6776866

[78] G. Qu, D. Peng, Z. Yu, X. Chen, X. Cheng, Y. Yang, T. Ye, Q. Lv, W. Ji, X. Deng, B. Zhou, Advances in the role of auxin for transcriptional regulation of lignin biosynthesis. Funct. Plant Biol. 48, 743–754 (2021). 33663680 10.1071/FP20381

[79] W. Jiang, Y. Xia, X. Su, Y. Pang, ARF2 positively regulates flavonols and proanthocyanidins biosynthesis in Arabidopsis thaliana. Planta 256, 44 (2022). 35857143 10.1007/s00425-022-03936-w

[80] Y. Li, S. Han, Y. Qi, Advances in structure and function of auxin response factor in plants. J. Integr. Plant Biol. 65, 617–632 (2023). 36263892 10.1111/jipb.13392

[81] X. Kong, C. Zhang, H. Zheng, M. Sun, F. Zhang, M. Zhang, F. Cui, D. Lv, L. Liu, S. Guo, Y. Zhang, X. Yuan, S. Zhao, H. Tian, Z. Ding, Antagonistic interaction between auxin and SA signaling pathways regulates bacterial infection through lateral root in Arabidopsis. Cell Rep. 32, 108060 (2020). 32846118 10.1016/j.celrep.2020.108060

[82] J.-W. Wang, L.-J. Wang, Y.-B. Mao, W.-J. Cai, H.-W. Xue, X.-Y. Chen, Control of root cap formation by microRNA-targeted auxin response factors in Arabidopsis. Plant Cell 17, 2204–2216 (2005). 16006581 10.1105/tpc.105.033076 PMC1182483

[83] S. Bergonzi, M. C. Albani, E. Ver Loren van Themaat, K. J. V. Nordström, R. Wang, K. Schneeberger, P. D. Moerland, G. Coupland, Mechanisms of age-dependent response to winter temperature in perennial flowering of Arabis alpina. Science 340, 1094–1097 (2013). 23723236 10.1126/science.1234116

[84] J. Gao, K. Zhang, Y.-J. Cheng, S. Yu, G.-D. Shang, F.-X. Wang, L.-Y. Wu, Z.-G. Xu, Y.-X. Mai, X.-Y. Zhao, D. Zhai, H. Lian, J.-W. Wang, A robust mechanism for resetting juvenility during each generation in Arabidopsis. Nat. Plants 8, 257–268 (2022). 35318444 10.1038/s41477-022-01110-4

[85] C.-M. Zhou, T.-Q. Zhang, X. Wang, S. Yu, H. Lian, H. Tang, Z.-Y. Feng, J. Zozomova-Lihová, J.-W. Wang, Molecular basis of age-dependent vernalization in *Cardamine flexuosa*. Science 340, 1097–1100 (2013). 23723237 10.1126/science.1234340

[86] Y. Zou, S. Wang, Y. Zhou, J. Bai, G. Huang, X. Liu, Y. Zhang, D. Tang, D. Lu, Transcriptional regulation of the immune receptor FLS2 controls the ontogeny of plant innate immunity. Plant Cell 30, 2779–2794 (2018). 30337428 10.1105/tpc.18.00297 PMC6305972

[87] T. Li, N. Gonzalez, D. Inzé, M. Dubois, Emerging connections between small RNAs and phytohormones. Trends Plant Sci. 25, 912–929 (2020). 32381482 10.1016/j.tplants.2020.04.004

[88] P. Singh, P. Dutta, D. Chakrabarty, miRNAs play critical roles in response to abiotic stress by modulating cross-talk of phytohormone signaling. Plant Cell Rep. 40, 1617–1630 (2021). 34159416 10.1007/s00299-021-02736-y

[89] E. Shen, T. Zhao, Q.-H. Zhu, Are miRNAs applicable for balancing crop growth and defense trade-off? New Phytol. 243, 1670–1680 (2024). 38952260 10.1111/nph.19939

[90] W.-H. Han, J.-X. Wang, F.-B. Zhang, Y.-X. Liu, H. Wu, X.-W. Wang, Small RNA and degradome sequencing reveal important microRNA function in *Nicotiana tabacum* response to *Bemisia tabaci*. Genes (Basel) 13, 361 (2022). 35205405 10.3390/genes13020361 PMC8871844

[91] T. Zhu, X. Zhou, J.-L. Zhang, W.-H. Zhang, L.-P. Zhang, C.-X. You, P. E. Jameson, P.-T. Ma, S.-L. Guo, Ethylene-induced *NbMYB4L* is involved in resistance against tobacco mosaic virus in *Nicotiana benthamiana*. Mol. Plant Pathol. 23, 16–31 (2022). 34633738 10.1111/mpp.13139 PMC8659562

