# Supplementary material for: Auxin-salicylic acid seesaw regulates the age-dependent balance between plant growth and herbivore defense
Source: Sci Adv. 2025 May 9;11(19):eadu5141. doi: 10.1126/sciadv.adu5141 (PMC12063664; doi:10.1126/sciadv.adu5141)
Supplement: Supplementary file 1 — Figs. S1 to S9 Tables S1 and S2 [file sciadv.adu5141_sm.pdf]

Science Advances
AAAS

Supplementary Materials for

Auxin-salicylic acid seesaw regulates the age-dependent balance between plant growth and herbivore defense

Wen-Hao Han *et al.*

Corresponding author: Xiao-Wei Wang, xwwang@zju.edu.cn

Sci. Adv. **11**, eadu5141 (2025)
DOI: 10.1126/sciadv.adu5141

This PDF file includes:

Figs. S1 to S9
Tables S1 and S2

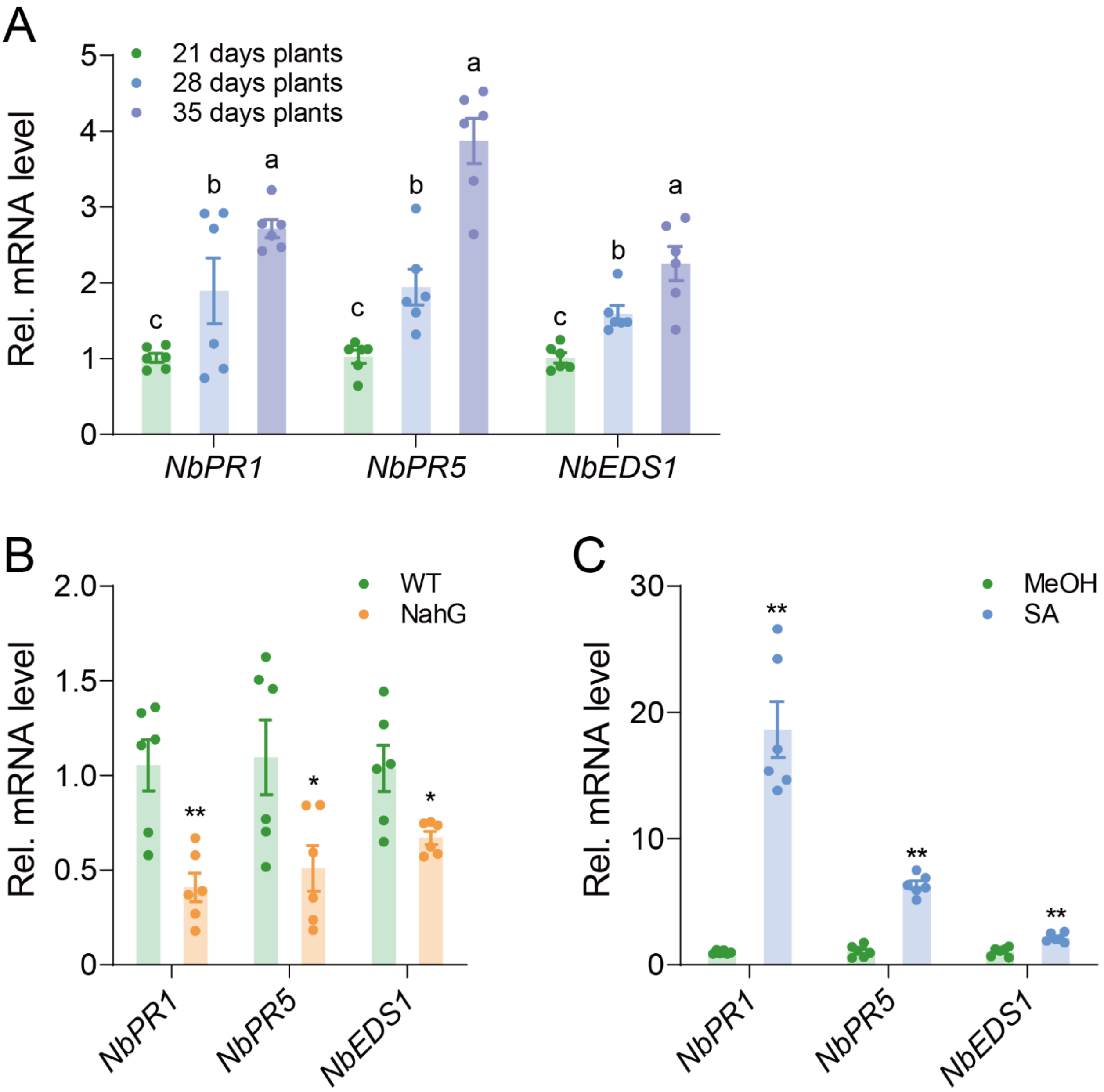

Fig. S1. Expression of the *NbPR1* gene downstream of SA at different ages and SA levels. (**A**) Expression level of the SA downstream genes *NbPR1*, *NbPR5*, and *NbEDS5*, in the *N. benthamiana* plants at 21, 28, and 35 days old. (**B**) Expression level of *NbPR1*, *NbPR5*, and *NbEDS5*, in 35-day-old wild-type and NahG *N. benthamiana* plants. (**C**) Expression level of *NbPR1*, *NbPR5*, and *NbEDS5*, in 21-day-old *N. benthamiana* plants treated with 1 mM SA. Values are mean ± SE, $n = 6$. Student's *t*-test (two-tailed) was used for significant difference analysis in **B** and **C**. *$P < 0.05$;**$P < 0.01$. One-way ANOVA with Fisher's least significant difference (LSD) test was used in **A**. Different lowercase letters indicate significant differences ($P < 0.05$).

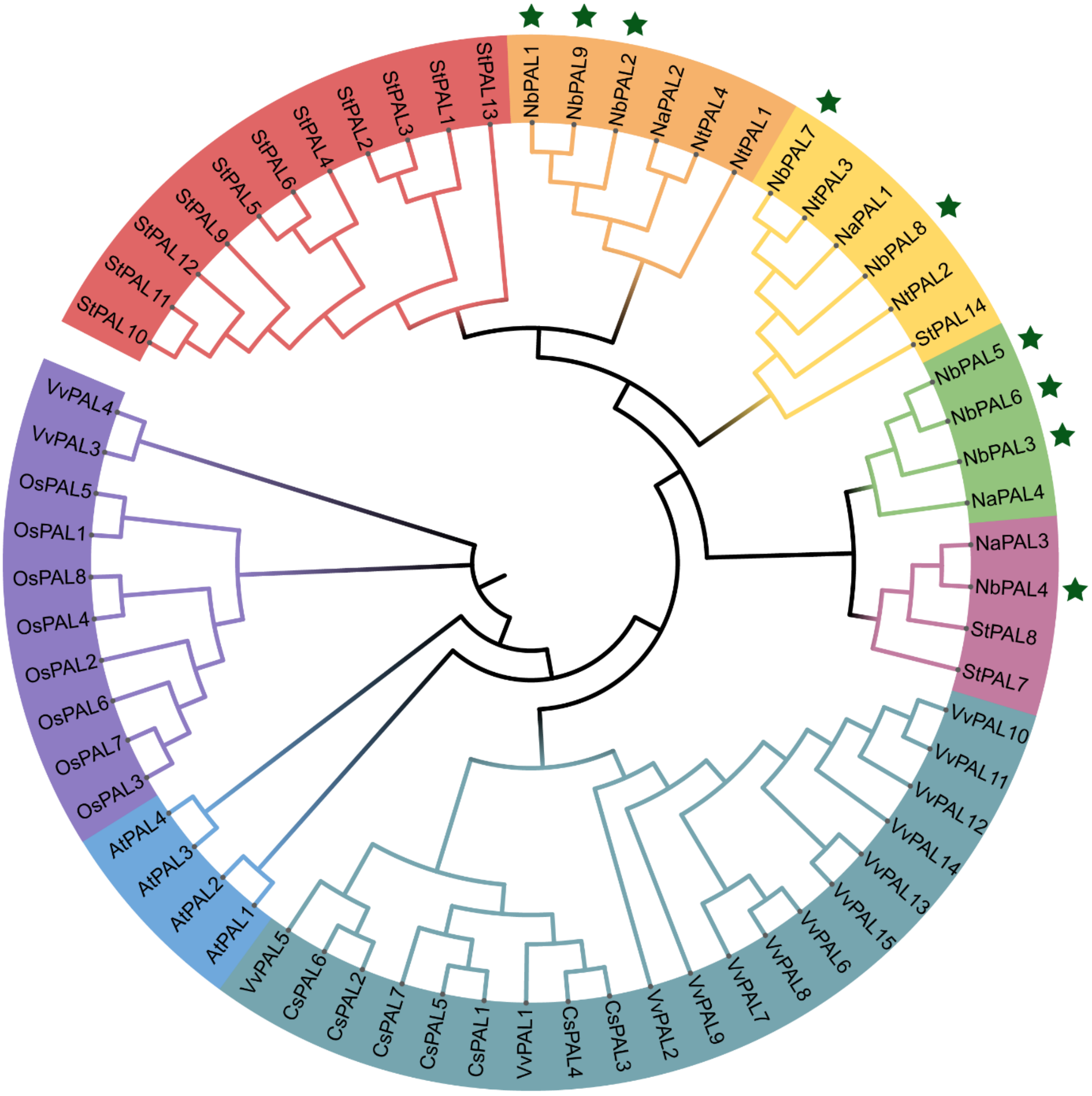

Fig. S2. Phylogenetic tree of PAL protein.

Amino acid sequences of PAL proteins from *N. benthamiana* (Nb), *N. tabacum* (Nt), *N. attenuata* (Na), *Arabidopsis thaliana* (At), *Oryza sativa* (Os), *Solanum tuberosum* (St), *Vitis vinifera* (Vv), and *Camellia sinensis* (Cs) were aligned by ClustalW. The Maximum Likelihood tree was constructed with MEGA X with 1000 bootstrap replicates.

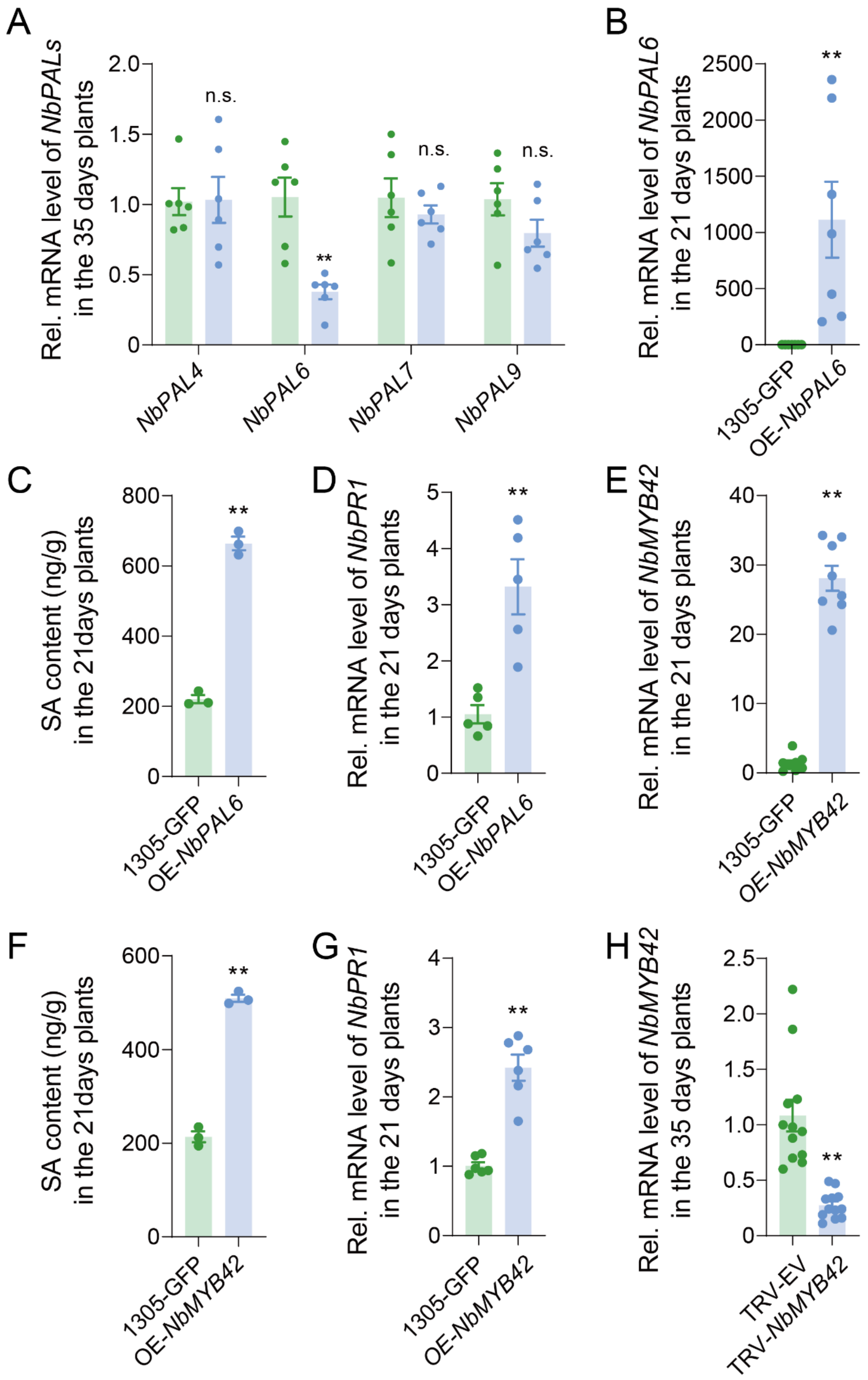

A
Rel. mRNA level of NbPALs in the 35 days plants
2.0
1.5
1.0
0.5
0
n.s.
**
n.s.
n.s.
NbPAL4
NbPAL6
NbPAL7
NbPAL9
B
Rel. mRNA level of NbPAL6 in the 21 days plants
2500
2000
1500
1000
500
0
**
1305-GFP
OE-NbPAL6
C
SA content (ng/g) in the 21days plants
800
600
400
200
0
**
1305-GFP
OE-NbPAL6
D
Rel. mRNA level of NbPR1 in the 21 days plants
5
4
3
2
1
0
**
1305-GFP
OE-NbPAL6
E
Rel. mRNA level of NbMYB42 in the 21 days plants
40
30
20
10
0
**
1305-GFP
OE-NbMYB42
F
SA content (ng/g) in the 21days plants
600
400
200
0
**
1305-GFP
OE-NbMYB42
G
Rel. mRNA level of NbPR1 in the 21 days plants
4
3
2
1
0
**
1305-GFP
OE-NbMYB42
H
Rel. mRNA level of NbMYB42 in the 35 days plants
2.5
2.0
1.5
1.0
0.5
0
**
TRV-EV
TRV-NbMYB42

Fig. S3. Gene expression level after silencing and overexpressing *NbPAL6* and *NbMYB42*.

(**A**) Expression level of *NbPAL4*, *NbPAL6*, *NbPAL7*, and *NbPAL9* in *NbPAL6*-silenced 35-day-old *N. benthamiana* plants. (**B**) Expression level of *NbPAL6* in *NbPAL6*-overexpressed 21-day-old *N. benthamiana* plants. (**C**) SA levels in the *NbPAL6*-overexpressed 21-day-old *N. benthamiana* plants. (**D**) Expression level of SA downstream *NbPR1* gene in *NbPAL6*-overexpressed 21-day-old *N. benthamiana* plants. (**E**) Expression level of *NbMYB42* in the *NbMYB42*-overexpressed 21-day-old *N. benthamiana* plants. (**F**) SA levels in the *NbMYB42*-overexpressed 21-day-old *N. benthamiana* plants. (**G**) Expression level of SA downstream *NbPR1* gene in the *NbMYB42*-overexpressed 21-day-old *N. benthamiana* plants. (**H**) Expression level of *NbMYB42* in *NbMYB42*-silenced 35-day-old *N. benthamiana* plants. Values are mean ± SE, $n = 6$ for **A** and **G**; $n = 7$ for **B**; $n = 3$ for **C** and **F**; $n = 5$ for **D**; $n = 8$ for **E**; $n = 12$ for **H**. Student's *t*-test (two-tailed) was used for significant difference analysis. n.s., not significant; $^{**}P < 0.01$.

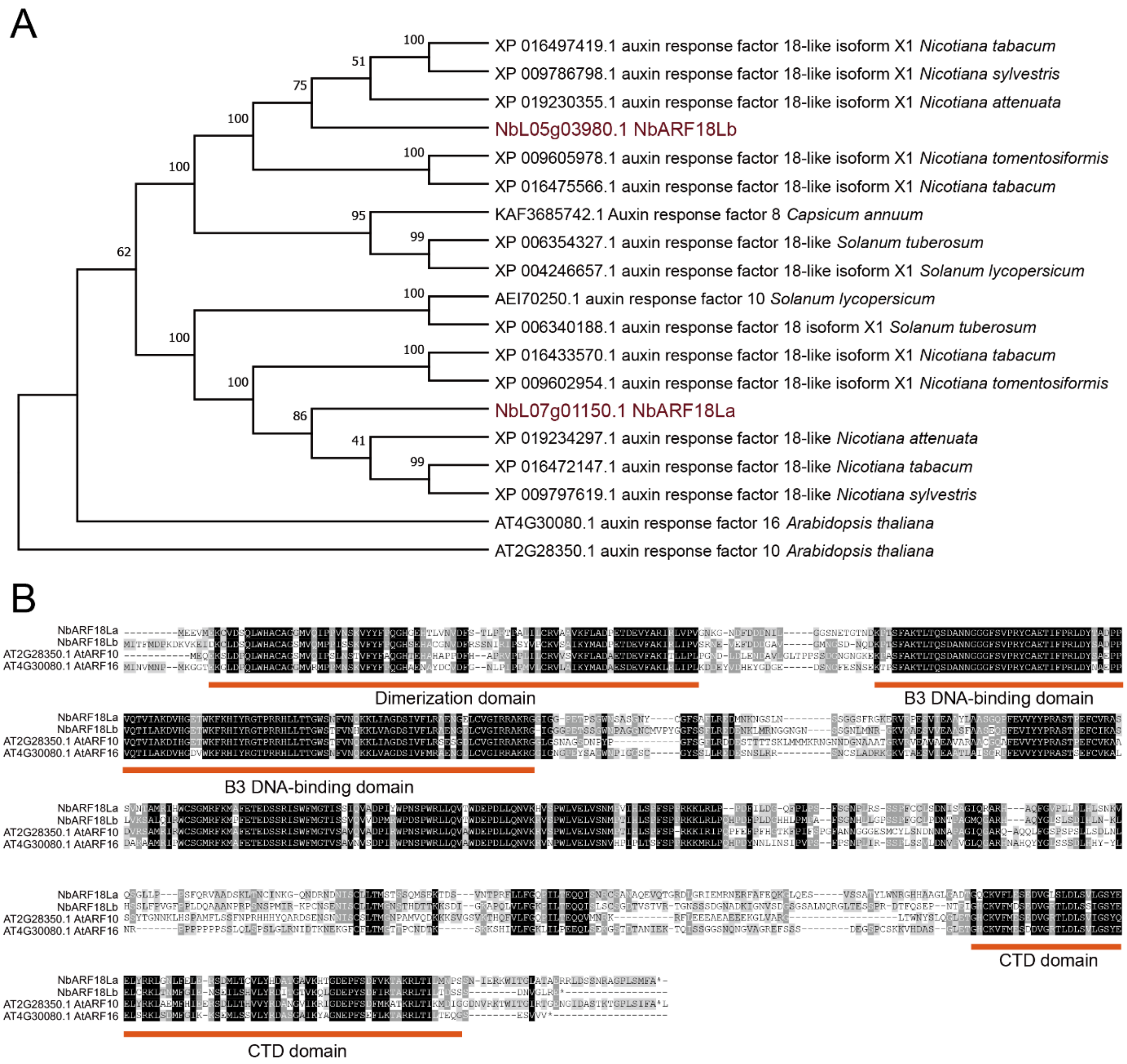

Fig. S4. Phylogenetic relationship and conserved DNA binding domain of NbARF18La/b. (**A**) Phylogenetic tree of ARF proteins. Homologous genes of *NbARF18Ls* were identified using NCBI Blast (https://blast.ncbi.nlm.nih.gov/Blast.cgi). Protein sequences were aligned using ClustalW, and a phylogenetic tree with 1000 bootstrap replicates was constructed by the Maximum Likelihood method in MEGA X. (**B**) Multi-sequence alignment analysis of ARF proteins. The B3 DNA binding domain (DBD) and C-terminal dimerization domain (CTD) regions are underlined. Protein sequence alignment results were visualized by GenDoc.

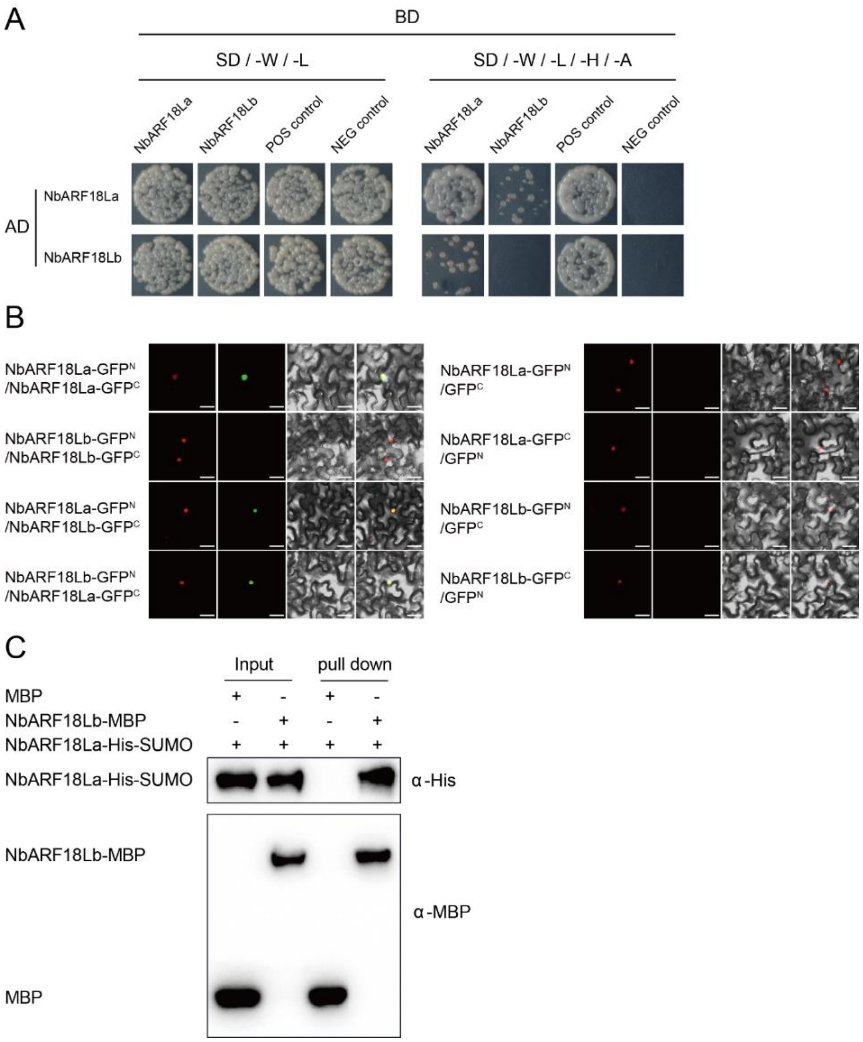

Fig. S5. NbARF18La interacts with NbARF18Lb.

(**A**) Yeast two-hybrid (Y2H) assay between NbARF18La and NbARF18Lb. Yeast cells co-transformed with NbARF18La and NbARF18Lb plasmids grow on a selective medium (SD/-W-L-H-A) for 3 days, indicating an interaction between NbARF18La and NbARF18Lb. Additionally, yeast cells co-transformed with NbARF18La plasmids (pGADT7-NbARF18La and pGBKT7-NbARF18La) also grew on selective medium (SD/-W-L-H-A), showing that NbARF18La exhibits self-interaction. (**B**) Bimolecular fluorescence complementation (BiFC) assays confirmed the interaction between NbARF18La and NbARF18Lb in *N. benthamiana* epidermal cells. BiFC assays show that NbARF18La interacts with NbARF18Lb in nuclei, and NbARF18La interacts with itself in nuclei. The BiFC signal (green fluorescence) observed in the nuclei indicates an interaction between NbARF18La and NbARF18Lb, as well as a self-interaction of NbARF18La. Confocal microscopy was used to visualize the fluorescence. Scale bars, 40 μm. (**C**) The interaction between NbARF18La and NbARF18Lb was further validated by an in vitro pull-down assay. Recombinant NbARF18Lb-MBP and NbARF18La-His-SUMO proteins were incubated with MBP beads in a pull-down binding buffer. The pulled-down proteins were detected by immunoblotting using anti-MBP and anti-His antibodies. A mixture of MBP and NbARF18La-His-SUMO was used as a negative control.

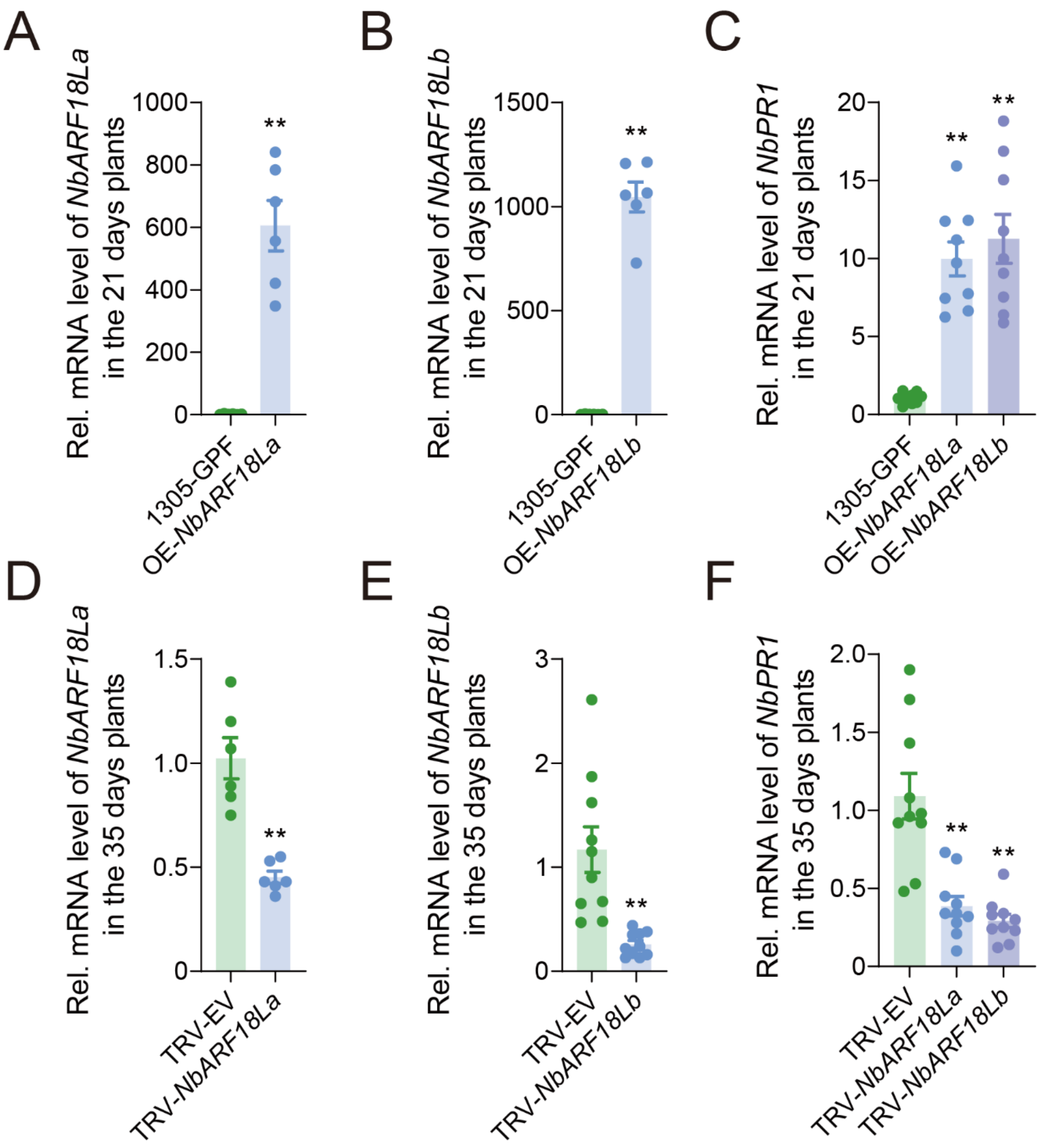

Fig. S6. Gene expression levels after overexpressing and silencing *NbARF18La/b.*

(**A**, **B**) Expression levels of *NbARF18La/b* in the *NbARF18La/b*-overexpressed 21-day-old *N. benthamiana* plants. (**C**) Expression level of SA downstream gene *NbPR1* in the *NbARF18La/b*-overexpressed 21-day-old *N. benthamiana* plants. (**D**, **E**) Expression levels of *NbARF18La* and *NbARF18Lb* in the *NbARF18La/b*-silenced 35-day-old *N. benthamiana* plants. (**F**) Expression level of SA downstream gene *NbPR1* in the *NbARF18La/b*-silenced 35-day-old *N. benthamiana* plants. Values are mean ± SE, $n = 6$ for **A**, **B**, and **D**; $n = 9$ for **C**; $n = 10$ for **E** and **F**. Student's *t*-test (two-tailed) was used for significant difference analysis. $^{**}P < 0.01$.

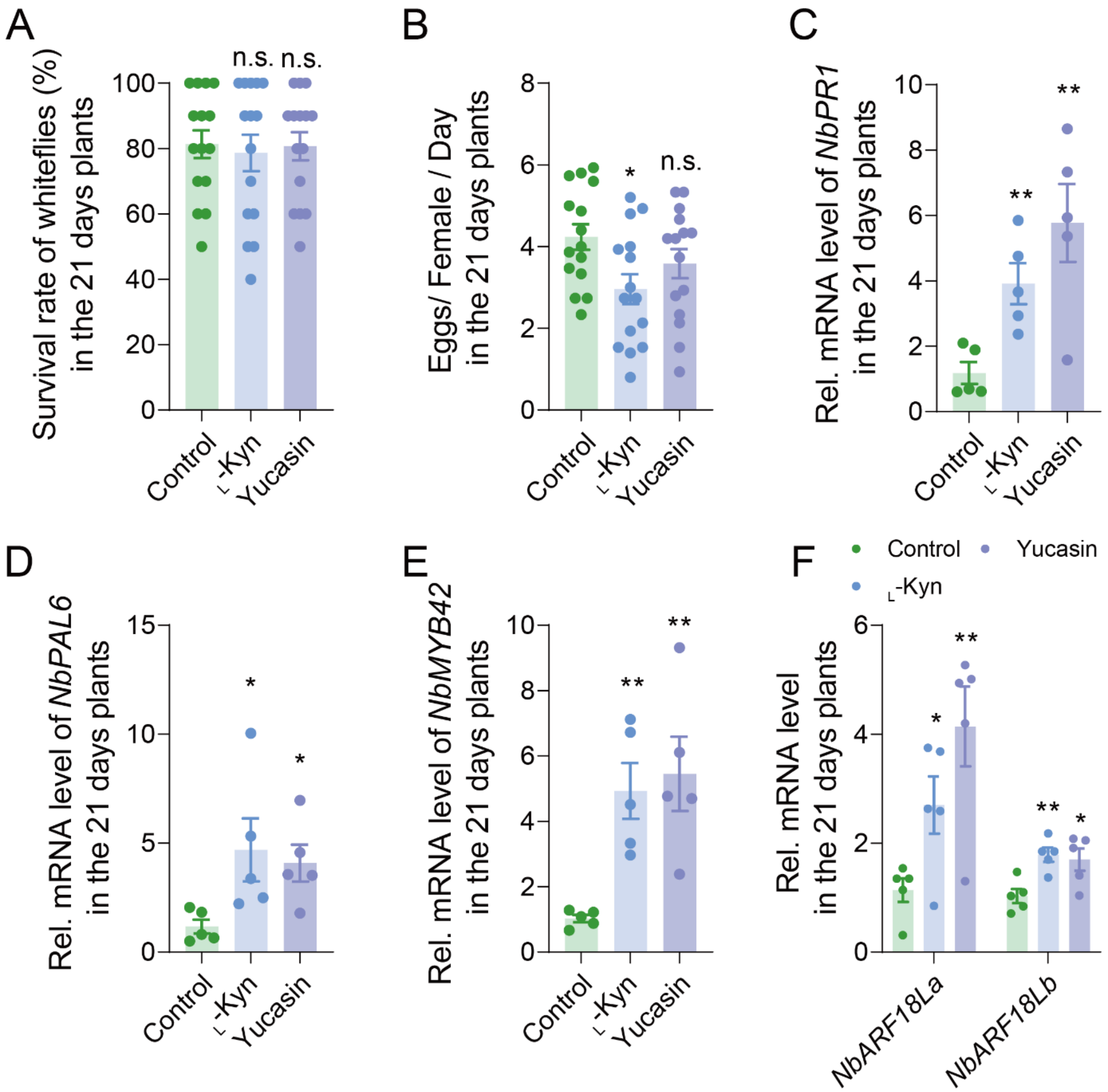

Fig. S7. Auxin synthesis inhibitors enhance the NbARF18La/b-NbMYB42 module in juvenile plants.

(**A**) Survival rate and fecundity of whiteflies on *N. benthamiana* plants treated with L-Kyn and Yucasin (50 μM) at 21 days old. (**B**) SA levels in *N. benthamiana* plants treated with the IAA inhibitors L-Kyn and Yucasin (50 μM) at 21 days old. (**C**) Expression level of SA downstream gene *NbPR1* in L-Kyn- and Yucasin-treated (50 μM) 21-day-old *N. benthamiana* plants. (**D**) Expression level of *NbPAL6* in L-Kyn- and Yucasin-treated (50 μM) 21-day-old *N. benthamiana* plants. (**E**) Expression level of *NbMYB42* in L-Kyn- and Yucasin-treated (50 μM) 21-day-old *N. benthamiana* plants. (**F**) Expression levels of *NbARF18La/b* in the L-Kyn- and Yucasin-treated (50 μM) 21-day-old *N. benthamiana* plants. Values are mean ± SE, *n* = 15 for **A** and **B**; *n* = 5 for **C**, **D**, **E**, and **F**. Student's *t*-test (two-tailed) was used for significant difference analysis. n.s., not significant; *$P < 0.05$, **$P < 0.01$.

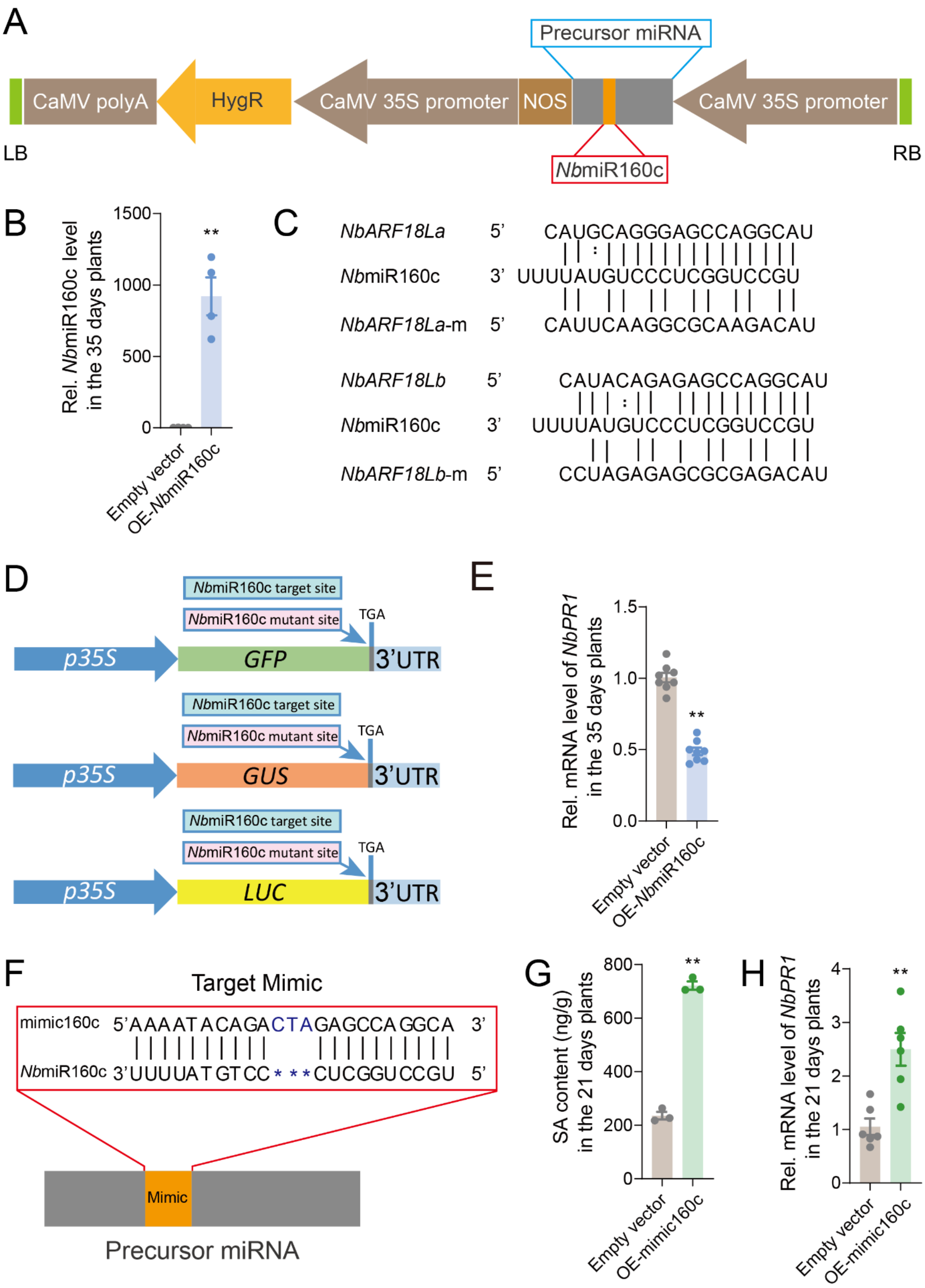
A
Precursor miRNA
CaMV polyA
HygR
CaMV 35S promoter
NOS
CaMV 35S promoter
LB
RB
NbmiR160c
B
Rel. NbmiR160c level in the 35 days plants
1500
1000
500
0
**
Empty vector
OE-NbmiR160c
C
NbARF18La 5' CAUGCAGGGAGCCAGGCAU
NbmiR160c 3' UUUUAUGUCCCUCGGUCCGU
NbARF18La-m 5' CAUUCAAGGCGCAAGACAU
NbARF18Lb 5' CAUACAGAGAGCCAGGCAU
NbmiR160c 3' UUUUAUGUCCCUCGGUCCGU
NbARF18Lb-m 5' CCUAGAGAGCGCGAGACAU
D
NbmiR160c target site
NbmiR160c mutant site
TGA
p35S
GFP
3'UTR
GUS
LUC
E
Rel. mRNA level of NbPR1 in the 35 days plants
1.5
1.0
0.5
0.0
**
Empty vector
OE-NbmiR160c
F
Target Mimic
mimic160c 5'AAAATACAGACTAGAGCCAGGCA 3'
NbmiR160c 3'UUUUATGTCC***CUCGGUCCGU 5'
Mimic
Precursor miRNA
G
SA content (ng/g) in the 21 days plants
800
600
400
200
0
**
Empty vector
OE-mimic160c
H
Rel. mRNA level of NbPR1 in the 21 days plants
4
3
2
1
0
**
Empty vector
OE-mimic160c

Fig. S8. Overexpression of mimicRNA160c increased the SA levels in juvenile plants.

(**A**) Schematic diagram of the *Nb*miR160c expression cassette *35S*-*Nb*miR160c. This vector was used to overexpress *Nb*miR160c in *N. benthamiana* plants through *Agrobacterium*-mediated transformation. The blue box represents the miRNA precursor, and the red box represents *Nb*miR160c. HygR: Hygromycin B phosphotransferase; NOS: Nos Terminator; RB: Right border; LB: Left border. (**B**) Expression level of *Nb*miR160c in the *Nb*miR160c-expressed 35-day-old plants. (**C**) Sequence alignment of *Nb*miR160c with the predicted target sites in *NbARF18La* and *NbARF18Lb*, along with their mutated versions (*NbARF18La*-m and *NbARF18Lb*-m) used in subsequent experiments. Colons represent G: U pairs. (**D**) Schematic diagram of the GFP/GUS/LUC sensor carrying the *Nb*miR160c target site and the mutant target site. TGA: Termination codon; UTR: Untranslated regions. (**E**) Expression level of *NbPR1* in the *Nb*miR160c-expressed 35-day-old plants. (**F**) Design of mimicry sequences (mimic160c) for *Nb*miR160c. (**G**) SA levels in mimic160c-expressed 21-day-old plants. (**H**) Expression level of *NbPR1* in the mimic160c-expressed 21-day-old plants. Values are mean ± SE, $n = 4$ for **B**; $n = 8$ for **E**; $n = 3$ for **G**; $n = 6$ for **H**. Student's *t*-test (two-tailed) was used for significant difference analysis. $^{**}P < 0.01$.

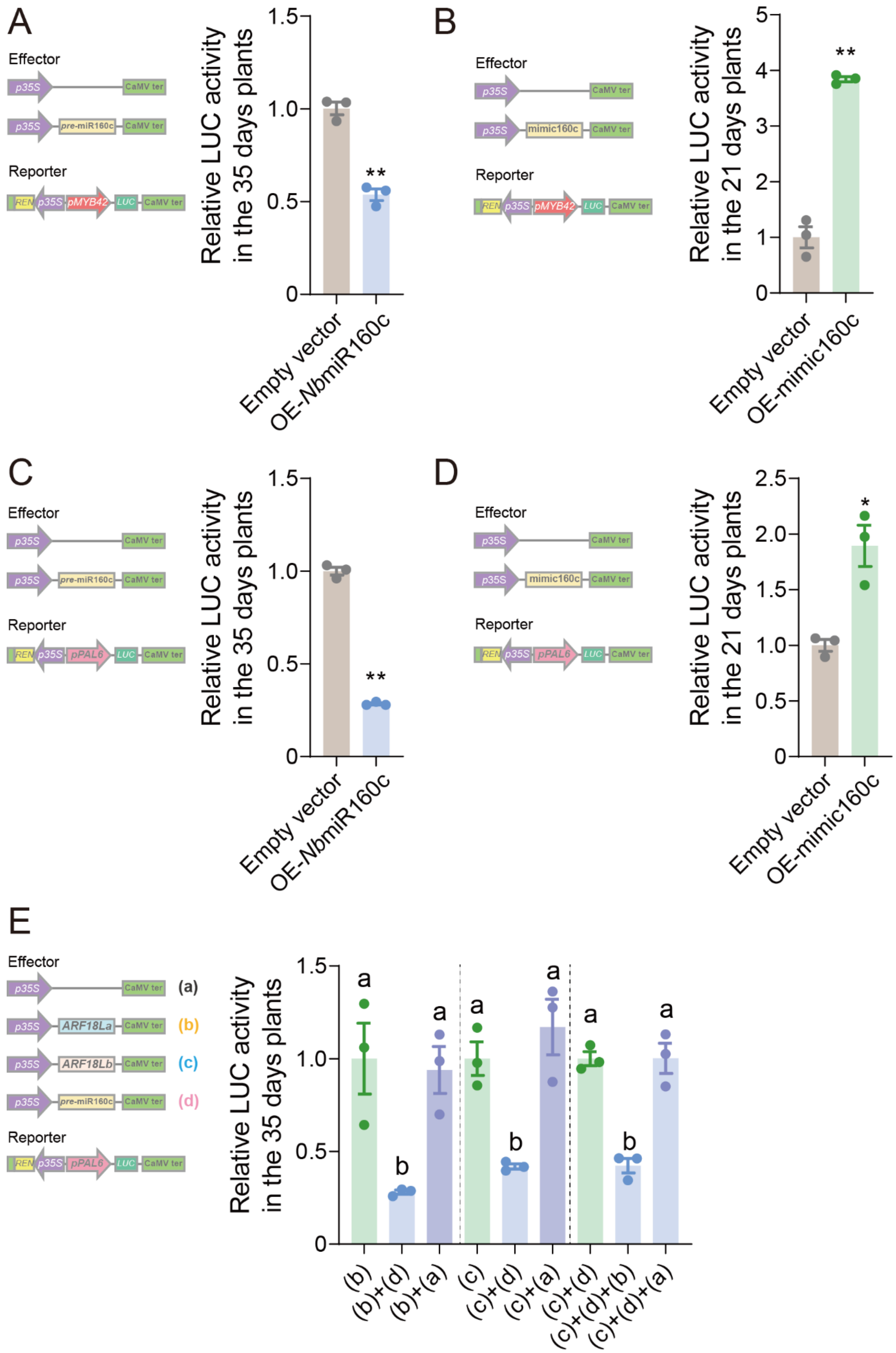

A
Effector
p35S
CaMV ter
pre-miR160c
Reporter
REN
pMYB42
LUC
Relative LUC activity in the 35 days plants
Empty vector
OE-NbmiR160c
**
B
mimic160c
Relative LUC activity in the 21 days plants
OE-mimic160c
C
pPAL6
D
*
E
ARF18La
ARF18Lb
(a)
(b)
(c)
(d)
(b)+(d)
(b)+(a)
(c)+(d)
(c)+(a)
(c)+(d)+(b)
(c)+(d)+(a)

Fig. S9. *Nb*miR160c affects the transcription of *NbMYB42* and *NbPAL6*.

(**A** to **E**) Schematic diagram showing the effector and reporter vectors used in transient transcriptional activity assays in *N. benthamiana* leaves. The data show the relative LUC activity normalized to REN activity. Values are mean ± SE, $n = 3$. Student's t-test (two-tailed) was used for significant difference analysis in **A**, **B**, **C**, and **D**. *$P < 0.05$, **$P < 0.01$. One-way ANOVA with Fisher's LSD test was used for significant difference analysis in **E**. Different lowercase letters indicate significant differences ($P < 0.05$).

Table S1. Basic information on identifying *NbPAL* genes.

Gene Name	Gene ID	CDS (bp)	Protein Length (aa)	Molecular Weight (Da)
NbPAL1	NbL01g24700.1	2040	679	73888.46
NbPAL2	NbL05g03880.1	2154	717	78005.01
NbPAL3	NbL10g04670.1	2154	717	78248.28
NbPAL4	NbL10g08440.1	2166	721	78734.94
NbPAL5	NbL12g19650.1	2154	717	78393.48
NbPAL6	NbL13g11330.1	2154	717	78233.31
NbPAL7	NbL14g10160.1	2139	712	77447.38
NbPAL8	NbL15g02170.1	2139	712	77438.39
NbPAL9	NbL15g24630.1	2154	717	78151.22

Table S2. List of primers used in this study.

Primer name	Sequence (5’-3’)
q-mir160c-F	TGCCTGGCTCCCTGTATTTT
q-NbU6-F	GGAACGATACAGAGAAGATTAGCA
q-NbACTIN-F	CAATCCAGACACTGTACTTTCTCTC
q-NbACTIN-R	AAGCTGCAGGTATCCATGAGACTA
q-NbPR1-F	TGCAAATCAGAGAGCTGGGG
q-NbPR1-R	CACTTTTCCCGGTGCACAAG
q-NbPR5-F	CCCGTTGGTTTTCAAGGCAG
q-NbPR5-R	TTGAGGGATGGACCGCAATC
q-NbEDS1-F	GTTGTGTCTTCGTGCAGCAG
q-NbEDS1-R	CACACGTGGTCTGGTACTCC
q-NbICS1-F	CGTGCTTATGGGGCAATTCG
q-NbICS1-R	ATTGCCATCTGGTACGTGCA
q-NbICS2-F	CTCGCGAGTACTCATGTCCC
q-NbICS2-R	ATCAATGTCCGCAGCTGTCA
q-NbCBP60g-F	CGACCTTGGCAAGCAGTTTC
q-NbCBP60g-R	GGAGAAACTGGCTGGATGCT
q-NbSARD1-F	AGTTGGCCACTGTTGACACA
q-NbSARD1-R	AGTGTAGTTTGCTCCGGTGG
q-NbEDS5-F	CAAACACTGCTGCTGCACAT
q-NbEDS5-R	AGGGACTTCAGCAACATCCG
q-NbPBS3-F	AACCCCGAAATTACCGACCC
q-NbPBS3-R	AGCACCCGTGACAATTACGT
q-NbEPS1-F	GCCGGCGTAGAATTCACCTA
q-NbEPS1-R	GCCGACGTTATTGAGTGGGA
q-NbMYB42-F	GGTGAGAGCTCCTTGTTGTGA
q-NbMYB42-R	TCTCCAATTGCCATGGCCAT
q-NbPAL4-F	ATGCACTCAAGGGTAGCCAC
q-NbPAL4-R	CAGCTCCACCATCACCTCTG
q-NbPAL6-F	AGGAACAAGGCATTGCATGG
q-NbPAL6-R	TTTCCAATGGAGGCAAGTGC
q-NbPAL7-F	ACAATCAGGGACAAGGGCAG
q-NbPAL7-R	GACCTGTGAGTAAACCGGCA
q-NbPAL9-F	TGTTTACCTCTCCGTGGCAC
q-NbPAL9-R	CAGCATTAAGGGCCTCACCA
q-NbARF18La-R	TGATCCCATCTACTGGCCCA
q-NbARF18La-F	GAGACCAGTTCGACAAGCCA
q-NbARF18Lb-F	CAACTCGTGCTTTTCGGCAA
q-NbARF18Lb-R	AGTGCAGAACCTGAGCCATC
q-NbTAR2-F	GCTGAGACTGGAAACCGACA
q-NbTAR2-R	CGAATAAAATGGGGCAGCGG

Primer name	Sequence (5’-3’)
q-NbYUC8-F	GCAAATGGCGCTGAAGTTGA
q-NbYUC8-R	TGCCAGAATTTCCACAGCCA
q-NbIAA29-F	CGTTTAAGGCTTGCGACGAG
q-NbIAA29-R	CCAATGGCCACTCCTTCCAT
pBI121-pNbPAL6-F	GACCATGATTACGCCAAGCTTGAATTCGTTCGGAAATTATGA
pBI121-pNbPAL6-R	ACCACCCGGGGATCCTCTAGAAAATGGAAAATATGAAAGAAAAAGA
pBI121-pNbARF18La-F	GACCATGATTACGCCAAGCTTCGCATGCAATCAAATATTATTACTCT
pBI121-pNbARF18La-R	ACCACCCGGGGATCCTCTAGATTGCCTTATCCAGTACACTTCT
pBI121-pNbARF18Lb-F	GACCATGATTACGCCAAGCTTGAGGTGAGGATTGCCCAAGC
pBI121-pNbARF18Lb-R	GGACTGACCACCCGGGGATCCCTTGATTTCAACAGAATTTTACAGA
pBI121-pNbMYB42-F	GCTATGACCATGATTACGCCAAGCTTTAGTGATTTGACTGTGCGTGA
pBI121-pNbMYB42-R	GACTGACCACCCGGGGATCCTCTAGATTTTGCTTTAATCACTTGCT
pBI121-pmir160-F	AGCTATGACCATGATTACGCCAAGCTTAATTTGACTATTACATAATTCCT
pBI121-pmir160-R	GGACTGACCACCCGGGGATCCCGAGGAAAATACGAAGTGAA
TRV-NbARF18La-F	GCTCTAGAACCCAGTTTCCAGCGAGTTG
TRV-NbARF18La-R	CGGGATCCTCTTGAAGACCTTTCTGCTCA
TRV-NbARF18Lb-F	GCTCTAGAAATCCAGCTGGTGGGAACTG
TRV-NbARF18Lb-R	CGGGATCCAAAAGGCATCTTGAACCTCATCC
TRV-NbPAL6-F	GCTCTAGATCTATGGTTCTATTTGAGGCT
TRV-NbPAL6-R	CGGGATCCGGCCCTAATGACCTCAATTTGA
TRV-NbMYB42-F	GAGTAAGGTTACCGAATTCTCTAGAAAAGACACTCCAAAAACAATCTTGA
TRV-NbMYB42-R	GTGAGCTCGGTACCGGATCCTTGGTCATCGATTGTAATAGTCGT
1305-NbARF18La-F	TAAGTCCGGAGCTAGCTCTAGAATGGAGGAGGTTATGGAGAAATGT
1305-NbARF18La-R	CGGTCCTCGAGACGTCTCTAGATGCAAACATGCTAAGTGGTCC
1305-NbARF18Lb-F	TAAGTCCGGAGCTAGCTCTAGAATGATTACTTTTATGGATCCAAAGGAC
1305-NbARF18Lb-R	CGGTCCTCGAGACGTCTCTAGACTCCCTGAGTCCAACATTATCG
1305-NbPAL6-F	GCTCTAGAATGGAGTATGCAAATGGAGATTGT
1305-NbPAL6-R	CGGGATCCGCAGAGTGGAAGAGGAGC

Primer name	Sequence (5’-3’)
1305-NbMYB42-F	GCTCTAGAATGGTGAGAGCTCCTTGTTGT
1305-NbMYB42-R	CGGGATCCAAATTCCGGTAAATCTAGT
pABAi-pNbMYB42-F	AAAAAAAATGATGAATTGAAAAGCTTTAGTGATTTGACTGTGCGTGA
pABAi-pNbMYB42-R	TCGAGGTCGACAGATCCCCGGGTACCTTTTGCTTTAATCACTTGCT
pABAi-pNbPAL6-F	AAAAAAAATGATGAATTGAAAAGCTTGAATTCGTTCGGAAATTATGA
pABAi-pNbPAL6-R	TCGAGGTCGACAGATCCCCGGGTACCAAATGGAAAATATGAAAGAAAAAGA
pGAD-NbARF18La-F	CCGGGTGGGCATCGATACGGGATCCATGGAGGAGGTTATGGAGAAATGT
pGAD-NbARF18La-R	TATCTACGATTCATCTGCAGCTCGAGCTATGCAAACATGCTAAGTGGTCC
pGAD-NbARF18Lb-F	CCGGGTGGGCATCGATACGGGATCCATGATTACTTTTATGGATCCAAAGGAC
pGAD-NbARF18Lb-R	TATCTACGATTCATCTGCAGCTCGAGTTACTCCCTGAGTCCAACAT
pGAD-NbMYB42-F	CGGGATCCATGGTGAGAGCTCCTTGTTGT
pGAD-NbMYB42-R	CCCTCGAGTCAAAATTCCGGTAAATCTAGT
pGBK-NbARF18La-F	TGGCCATGGAGGCCGAATTCATGGAGGAGGTTATGGAGAAATGT
pGBK-NbARF18La-R	CGCTGCAGGTCGACGGATCCCTATGCAAACATGCTAAGTGGTCC
pGBK-NbARF18Lb-F	TGGCCATGGAGGCCGAATTCATGATTACTTTTATGGATCC
pGBK-NbARF18Lb-R	CGCTGCAGGTCGACGGATCCCTACTCCCTGAGTCCAACAT
pGreen0800-pNbPAL6-F	TCGAGGTCGACGGTATCGATAAGCTTGAATTCGTTCGGAAATTATGA
pGreen0800-pNbPAL6-R	GGCCGCTCTAGAACTAGTGGATCCAAATGGAAAATATGAAAGAAAAAGA
pGreen0800-pNbMYB42-F	TCGAGGTCGACGGTATCGATAAGCTTTAGTGATTTGACTGTGCGTGA
pGreen0800-pNbMYB42-R	GCGGCCGCTCTAGAACTAGTGGATCCTTTTGCTTTAATCACTTGCT
pGreen0800-pmir160-F	ACTCACTATAGGGCGAATTGGGTACCAATTTGACTATTACATAATTCCT
pGreen0800-pmir160-R	CGGCCGCTCTAGAACTAGTGGATCCCGAGGAAAATACGAAGTGAA
pET28a-SUMO-NbARF18La-F	GCTCACAGAGAACAGATTGGTGGAATGGAGGAGGTTATGGAGAAATGTG
pET28a-SUMO-NbARF18La-R	TGCCGCCACTACCACCACCACTACCACCGCCTTTTTCAAACTGCGGATGGCTCCATGCAAACATGCTAAGTGGTCCTGC
pMAL-NbARF18Lb-F	TCCAGGGGCCCCTGGGATCCATGATTACTTTTATGGATCC

Primer name	Sequence (5’-3’)
pMAL-NbARF18Lb-R	CTGCCGCCACTACCACCACCACTACCACCGCCTTTTTCAAACTGCGGATGGCTCCACTCCCTGAGTCCAACATTATCGC
pGEX-NbMYB42-F	CTGGAAGTTCTGTTCCAGGGGCCCGGCTCCATGGTGAGAGCTCCTTGTTGTGA
pGEX-NbMYB42-R	CACTACCACCACCACTACCACCGCCTTTTTCAAACTGCGGATGGCTCCAAAATTCCGGTAAATCTAGTAAGTCCCCG
1300-mir160-F	GGGGTACCCAGCAGCAGCCACAGCAAAA
1300-mir160-R	CGGGATCCGCTGCTGATGCTGATGCCAT
1300-mimic160-F	GGTACCTGGCCATCCCCTA
1300-mimic160-R	GGATCCCGGAAGCAAATTTACA
Bifc-NbARF18La-F	CCATTTACGAACGATAGTTAATTAACATGGAGGAGGTTATGGAGAAATGT
Bifc-NbARF18La-R	CCACCACTGCCACCTCCTCCACTAGTTGCAAACATGCTAAGTGGTCC
Bifc-NbARF18Lb-F	CCATTTACGAACGATAGTTAATTAACATGATTACTTTTATGGATCCAAAGGAC
Bifc-NbARF18Lb-R	CCACCACTGCCACCTCCTCCACTAGTCTCCCTGAGTCCAACATTATCG
35S-GFP-mir160-F	AGGACCGGTCCCGGGGGATCCATGGTGAGCAAGGGCGAG
GFP-NbARF18La-site-R	GGGCGGCCGCTTTAAGATCTATGCCTGGCTCTCTGTATGC
GFP-NbARF18La-mutant site-R	GGGCGGCCGCTTTAAGATCTATGTCTCGCGCTCTCTAGGC
GFP-NbARF18Lb-site-R	GGGCGGCCGCTTTAAGATCTATGCCTGGCTCCCTGCAT
GFP-NbARF18Lb-mutant site-R	GGGCGGCCGCTTTAAGATCTATGTCTTGCGCCTTGAATGC
35S-GUS-mir160-F	GGAGAGAACACGGGGGACTCTAGAATGTTACGTCCTGTAGAAACCCC
GUS-NbARF18La-site-R	AACGATCGGGGAAATTCGAGCTCTCAATGCCTGGCTCTCTGTATGCC
GUS-NbARF18La-mutant site-R	AACGATCGGGGAAATTCGAGCTCTCAATGTCTCGCGCTCTCTAGGCC
GUS-NbARF18Lb-site-R	AACGATCGGGGAAATTCGAGCTCTCAATGCCTGGCTCCCTGCATGCC
GUS-NbARF18Lb-mutant site-R	AACGATCGGGGAAATTCGAGCTCTCAATGTCTTGCGCCTTGAATGCC
35S-LUC-mir160-F	CATGCCATGGAAGACGCCAA
LUC-NbARF18La-site-R	GCTCTAGAATGCCTGGCTCTCTGTATGCCATTACACGGCGATCTTTCCG
LUC-NbARF18La-mutant site-R	GCTCTAGAATGTCTCGCGCTCTCTAGGCCATTACACGGCGATCTTTCCG
LUC-NbARF18Lb-site-R	GCTCTAGAATGCCTGGCTCCCTGCATGCCATTACACGGCGATCTTTCCG

Primer name	Sequence (5'-3')
LUC-NbARF18Lb-mutant site-R	GCTCTAGAATGTCTTGCGCCTTGAATGCCATTACACGGCGATCTTTCCG
ChIP-pNbMYB42-P1-F	AGTGATTTGACTGTGCGTGA
ChIP-pNbMYB42-P1-R	AAAACGGCACAAATGGTCCG
ChIP-pNbMYB42-P2-F	AACTTGAGAACCCACGACCC
ChIP-pNbMYB42-P2-R	CCTGCATGGAAATTCATGAAATCT
ChIP-pNbMYB42-P3-F	ACTCCTTCAGATTTTGACGTGA
ChIP-pNbMYB42-P3-R	ACCTTATTTACGAGGATTACACTGAGT
ChIP-pNbPAL6-P1-F	TCGGTCTCAACGCGTTAATGA
ChIP-pNbPAL6-P1-R	ACTTTCCCTTCACCAACATGA
ChIP-pNbPAL6-P2-F	ATGACGTGGTGCTGTCGTTG
ChIP-pNbPAL6-P2-R	TTTCCCTCGTAAAATACCGCA
ChIP-pNbPAL6-P3-F	CGCCAGTAAGTTATTATCGCGC
ChIP-pNbPAL6-P3-R	CGTTGGATTGCACATGGAGA
ChIP-pNbPAL6-P4-F	TCTCCATGTGCAATCCAACG
ChIP-pNbPAL6-P4-R	TGTTGAGCCAAAATGTGAGTGT
ChIP-pNbPAL6-P5-F	TGGCTCAACAAACTCAGGAAATT
ChIP-pNbPAL6-P5-R	AATGTGTGTTGCGAACTTCTC
EMSA-pPAL6-F	GTGAGGGTTGAAGGGTGGTTGTTGAGAGGAAGCCCGCTTACTTTTTTTGG
EMSA-pPAL6-R	GGTTTTTTTCATTCGCCCGAAGGAGAGTTGTTGGTGGGAAGTTGGGAGTG
EMSA-pPAL6-mF	GTGAGGGTTGAAGAAAAAAAAAAAAAAAAAAAGCCCGCTTACTTTTTTTGG
EMSA-pPAL6-mR	GGTTTTTTTCATTCGCCCGAAAAAAAAAAAAAAAAAAAGAAGTTGGGAGTG
EMSA-pMYB42-F	GGACAATTTTTCTTTATTTTCTTTTTTGTCTCCAGTAAAATAACG
EMSA-pMYB42-R	GCAATAAAATGACCTCTGTTTTTTCTTTTATTTCTTTTTAACAGG
EMSA-pMYB42-mF	GGACAATTTTTCTTTATTTTCTTTTTAAAAAACAGTAAAATAACG
EMSA-pMYB42-mR	GCAATAAAATGACAAAAAATTTTTCTTTTATTTCTTTTTAACAGG
